# Rheumatoid arthritis and osteoporosis: a bi-directional Mendelian randomization study

**DOI:** 10.18632/aging.203029

**Published:** 2021-05-18

**Authors:** Ying-Qi Liu, Yong Liu, Zhuo-Yuan Chen, Hui Li, Tao Xiao

**Affiliations:** 1 Department of Orthopedics, The Second Xiangya Hospital of Central South University, Changsha 410011, China; 2 Center for System Biology, Data Sciences, and Reproductive Health, School of Basic Medical Science, Central South University, Changsha 410011, China

**Keywords:** osteoporosis, rheumatoid arthritis, genome-wide association study, Mendelian randomization

## Abstract

Many observation studies have demonstrated a close relationship between rheumatoid arthritis (RA) and osteoporosis (OP). However, the causal genetic correlation between RA and OP remains unclear. In this study, we performed bi-directional Mendelian randomization (MR) analyses to explore causal inference between these two traits. The instrumental variables for RA were selected from a large-scale genome-wide association study (GWAS) (1,523 cases and 461,487 controls). Bone mineral density (BMD) at five different sites (heel (n=265,627), forearm (FA) (n=8,143), femoral neck (FN) (n=32,735), lumbar spine (LS) (n=28,498), and total body (n=28,498)) were used as phenotypes for OP. The inverse variance weighted (IVW) method did not detect any causal effect of BMDs on RA except heel BMD (beta = -7.57 × 10-4, p = 0.02). However, other methods (MR-Egger, weighted median, weighted mode, MR-PRESSO, and MR-RAPS) showed no causal association between heel BMD and RA. Likewise, we did not find a causal effect of RA on BMD at any sites. In conclusion, we found no evidence that RA is causally associated with OP/BMD, or vice versa. We suggested that the associations found in previous observational studies between RA and OP/BMD are possibly related to secondary effects such as antirheumatic treatment and reduced physical activity.

## INTRODUCTION

Rheumatoid arthritis (RA) is a kind of common autoimmune disease with hyperplasia of joint tissue and synovial inflammation that can finally lead to some serious systematic disorders, such as cardiovascular, pulmonary, skeletal disorders, and psychological [1]. It affects around 1.3 million people in the USA [2], and 0.32%-0.36% population in China [3]. One of the most severe comorbidities of RA is osteoporosis (OP), which is a chronic metabolic skeletal disease leading to an increased risk of low trauma fracture [4]. Osteoporosis can be characterized by microarchitectural deterioration of bone tissue and low bone mass. Epidemiology studies indicate that about 60-80% of RA patients have a comorbidity of OP [5]. The most commonly used measurement for OP is bone mineral density (BMD) [6].

Both RA and OP have a strong genetic component. Previous studies have suggested that the heritability of RA is approximately 60% [7], while the heritability of OP is up to 50-85% [8]. To date, genome-wide association studies (GWASs) have successfully identified more than 200 single-nucleotide polymorphisms (SNPs) for OP and explained approximately 5% of the genetic heritability [9]. GWASs for RA have identified more than 100 susceptibility loci and explained approximately 12% of the genetic heritability [10]. These two kinds of complex diseases may share some common genetic mechanisms and biological processes. For example, proinflammatory cytokines including TNF-α, IL-17, IL-6, and IL-1 have been reported to be closely associated with OP [11], and they also play important roles in the development of RA [12].

In addition, many observation studies demonstrate a close relationship between OP and RA with strong evidence. Focal or generalized bone involvement occurs frequently in RA patients. Güler-Yüksel M et al. reported that osteoporosis was found in the spine and hip in 11% and 25% of 381 recently diagnosed active RA patients [13]. Synovial membrane inflammation will lead to periarticular cortical bone loss and marginal bone erosion, while generalized osteoporosis involving the appendicular and axial skeletons probably occurs before the onset of articular disease [14]. Follow-up studies conducted by the same group demonstrated that joint damage and joint damage progression were independently associated with high bone mineral density (BMD) loss both in hip/spine and hands after 1 year of treatment [15]. Longer duration and severity of RA were also indicated as independent risk factors for vertebral fractures [16].

**Figure 1:**
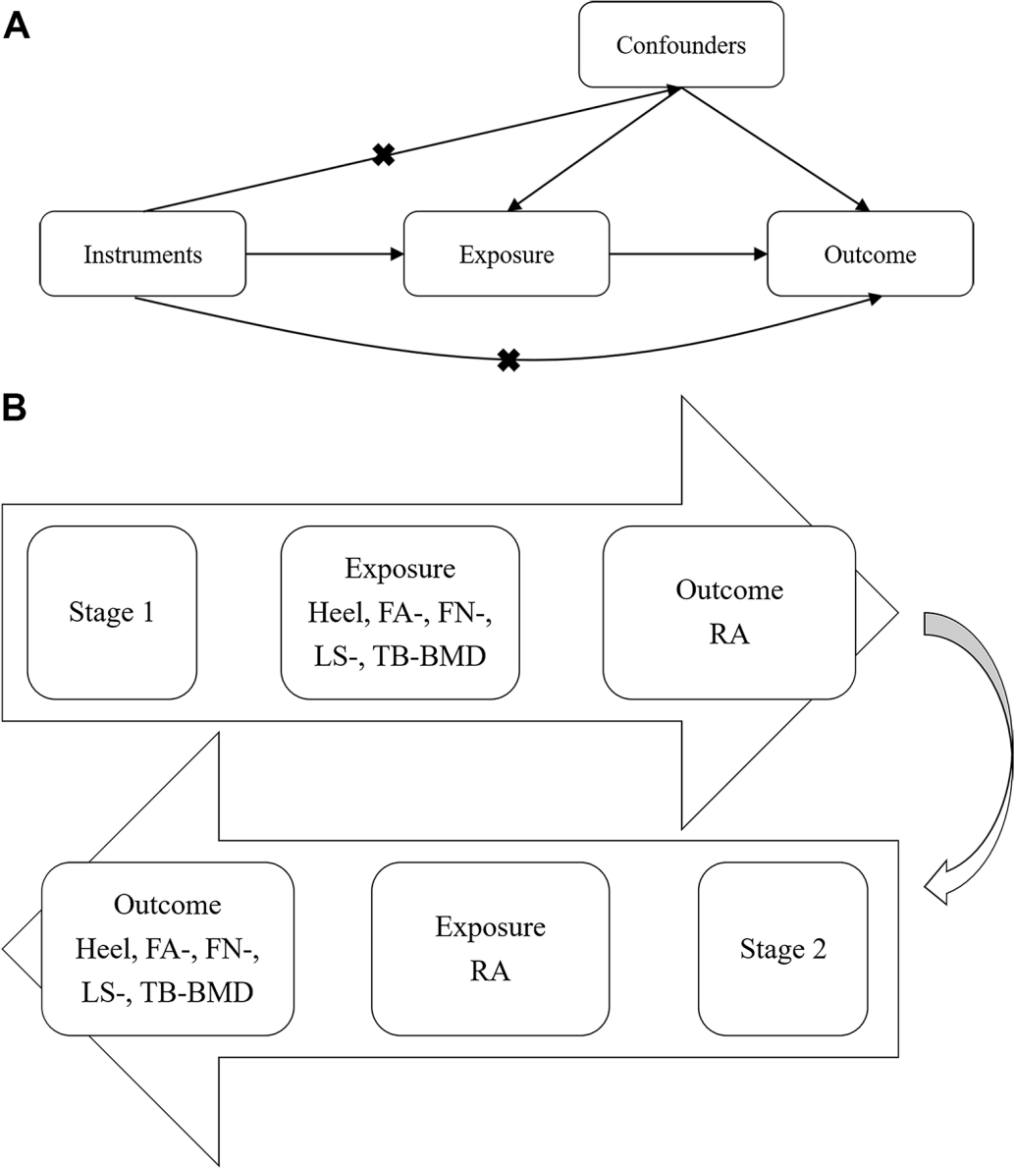
**Workflow of bi-directional MR analysis.** (**A**) The fundamental idea of MR analysis: If we cannot randomize the exposure, we can find a randomized instrumental variable to disentangle (**B**) Workflow of our bi-directional MR analysis. MR: Mendelian randomization; BMD: Bone mineral density; RA: Rheumatoid arthritis.

Does RA have a direct effect on OP, or vice versa? Due to the potential bias introduced by confounding factors, these prior observation data were limited for causal inference. The gold standard method for identifying causality is randomized controlled trial (RCT) design, but it consumes considerable time and money. In recent years, Mendelian randomization (MR) has been widely performed as an alternative method to assess causal relationships in observational data [17]. Genetic variants are used as instrumental variables (IVs) in the MR method to leverage the random assortment of genetic variants during gamete formation. If we assume that there are no genetic mating restrictions upon the population (panmixia), then the genotype distribution of this population should be unrelated to the confounding factors that typically affect observational epidemiology studies. In this regard, MR can be thought of as a “natural” RCT. Moreover, the assignment of genotypes is not affected by age, sex, lifestyle, or other environmental factors. Hence, the greatest advantage of MR is avoiding the effect of potential confounders or reverse causality compared with the general RCT design [18]. A genetic variant can be considered as an instrumental variable for a given exposure if it satisfies the 3 assumptions: 1) They are strongly associated with exposure. 2) They are independent of any known confounders. 3) They are conditionally independent of given exposure, outcome, and potential confounders, meaning that it does not affect the outcome except via the exposure, and it is not associated with the outcome due to confounding (Figure 1A).

Two-sample MR uses GWAS summary statistical data of both exposure and outcome traits to infer the causal association between exposure and outcome. Hence, it is not necessary to obtain the effect of the instrumental variable-exposure/-outcome association from the same sample of participants. In other words, two-sample MR allows us to perform MR between two traits using only independent GWAS summary statistics. In addition, there are some advantages to obtain summary statistical data from two different groups of participants. For example, the “winners’ curse” is unlikely to happen in two-sample MR, while it can underestimate true causal effects in one-sample MR [19]. Likewise, the weak instrument bias that biases effects towards the confounded multivariable regression result has a great impact in one-sample MR, but it is towards null in two-sample MR. The main advantage of using summary data from large GWASs in two-sample MR is the increasing of statistical power, particularly in testing effects on binary disease outcomes. Regardless of many successes of MR in investigating the potential causality of environmental factors-to-diseases or diseases-to-diseases, whether there is a causal effect between OP and RA is still unclear, though extensive evidence from observational studies showed a strong correlation between these two diseases. Therefore, we performed bi-directional MR analyses for causal inference between RA and OP in the present study. Since BMD is the most often used measurement for diagnosing OP [20, 21], summarized GWAS data for five different BMD measurements were included: the 1^st^~3^rd^ BMD measurements were DXA measuring-based BMD at forearm (FA), low spine (LS), and femoral neck (FN), which were most commonly used for diagnosing of OP and OP-related fracture [22]; the 4^th^ BMD measurement is DXA measuring-based BMD of total body, which has been reported with a strong correlation with BMD at low spine (LS) and femoral neck (FN), and with the largest collection of DXA measuring-based BMD [23]; the 5^th^ BMD measurement is heel BMD measured by quantitative heel ultrasounds from UK biobank [24]. Although the heel BMD estimated from quantitative heel ultrasounds is not as standard as DXA-based measurements, it is also strongly associated with DXA-based BMD. In addition, benefit from its convenience and low cost, the study of eBMD in UK biobank is much larger than any DXA-based study.

The bi-directional MR study design is shown in Figure 1B. In the first stage, we examined whether BMD measurements have causal effects on RA. In the second stage, we detected whether RA is causally associated with BMD measurements.

## RESULTS

### Stage1: Influence of BMD traits on RA

Respectively, we obtained 342, 3, 14, 14 and 50 IVs without effects of linkage disequilibrium (LD, r^2^ < 0.001) that reached genome-wide significance level (p < 5 × 10^-8^) from GWASs for heel, FA-, FN-, LS- and TB-BMD (Supplementary Table 1). The heterogeneity test showed no significant heterogeneity among selected IVs (Q_p value > 0.05, Table 1), except IVs of FN-BMD (Q_IVW = 24.73, Q_pval_IVW=0.02). All tests for MR Egger regression and leave-one-out analysis were negative (p for MR-Egger intercept > 0.05) (Table 1 and Supplementary Figures 1–5), indicating that our MR results were not biased by heterogeneity or horizontal pleiotropy. To demonstrate the power of selected IVs, we presented F statistics. The values of F statistics for selected IVs and the variance explained by them for heel BMD, FA-BMD, FN-BMD, LS-BMD, and TB-BMD were 97.05, 58.20, 52.33, 58.39, and 66.32, respectively. All of the F statistics were larger than 10, demonstrating that the selected IVs were strong enough to decrease any potential bias of the causal analyses. As expected, the negative control analyses presented that heel BMD, FA-BMD, FN-BMD, LS-BMD, and TB-BMD were not associated with myopia, suggesting that IVs of exposures we selected for this study were appropriate (Supplementary Tables 2, 3).

**Table 1:** Mendelian randomization estimates for BMD on RA.

**Exposure**	**Outcome**	**No. of IVs**	**Heterogeneity tests**		**Directional horizontal pleiotropy test**	**MR results**		
			**Methods**	**Cochran's Q (p)**	**MR-Egger intercept (p)**	**Method**	**Beta**	**P**
**Heel BMD**	RA	342	MR Egger	370.51 (0.12)	-1.87e-05 (0.20)	Inverse variance weighted	-7.57E-04	0.02
			Inverse variance weighted	372.28 (0.12)		MR Egger	-1.48E-05	0.98
						Weighted median	-2.48E-04	0.62
						Weighted mode	6.49E-05	0.93
						MR-Presso	-5.44E-04	0.08
						MR-Raps	-5.49E-04	0.07
**FA-BMD**	RA	3	MR Egger	2.94 (0.09)	-2.36e-04 (0.65)	Inverse variance weighted	-5.64E-04	0.49
			Inverse variance weighted	4.08 (0.13)		MR Egger	1.16E-03	0.76
						Weighted median	-1.87E-04	0.78
						Weighted mode	-7.27E-05	0.93
**FN-BMD**	RA	14	MR Egger	18.26 (0.11)	-5.13e-04 (0.06)	Inverse variance weighted	-3.03E-04	0.70
			Inverse variance weighted	24.74 (0.02)		MR Egger	-9.08E-03	0.06
						Weighted median	-3.40E-04	0.67
						Weighted mode	1.17E-04	0.93
**LS-BMD**	RA	14	MR Egger	19.92 (0.07)	-2.36e-04 (0.41)	Inverse variance weighted	-1.16E-04	0.86
			Inverse variance weighted	21.15 (0.07)		MR Egger	-3.66E-03	0.40
						Weighted median	-3.24E-04	0.65
						Weighted mode	-5.09E-04	0.65
**TB-BMD**	RA	50	MR Egger	50.46 (0.38)	9.29e-05 (0.09)	Inverse variance weighted	-3.49E-04	0.35
			Inverse variance weighted	53.64 (0.30)		MR Egger	-2.08E-03	0.06
						Weighted median	-6.23E-04	0.25
						Weighted mode	-6.54E-04	0.38

MR-PRESSO and MR-RAPS were performed for pairs of exposure-outcomes with more than 100 IVs. BMD: bone mineral density; RA: rheumatoid arthritis; FA: forearm; FN: femoral neck; LS: lumbar spine; TB: total body; IVs: instrumental variables; MR-PRESSO: Mendelian Randomization Pleiotropy RESidual Sum and Outlier; MR-RAPS: Mendelian Randomization Robust Adjusted Profile Score.

The inverse variance weighted (IVW) method supported a causative association between heel BMD and RA (beta = -7.57 × 10^-4^, p = 0.02), but MR Pleiotropy RESidual Sum and Outlier (MR-PRESSO) did not detect any potential pleiotropic IVs for BMD, and the corrected MR causal association between BMD and RA was negative (beta = -5.44 × 10^-4^, p = 0.08) (Table 1). As there were 342 IVs for heel BMD, we performed MR Robust Adjusted Profile Score (MR-RAPS) to test whether BMD affects RA through many weak instruments (beta = -5.49 × 10^-4^, p = 0.07) (Table 1). MR-RAPS did not show that heel BMD has a causal effect on RA. Furthermore, IVW analysis showed that FA-, FN-, LS- and TB-BMD were all negatively associated with RA (beta range from -1.16 × 10^-4^ to -5.64 × 10^-4^). In addition, MR-Egger, weighted median, and weighted mode methods did not identify any causal effect of heel, FA-, FN-, LS- and TB-BMD on RA (Table 1). Combined with results from different MR methods, we concluded that BMD has no causal effect on RA.

### Stage2: Influence of RA on BMD traits

In total, we obtained 6 LD-independent (r^2^ < 0.001) instrumental variables (IVs) with p < 1 × 10^-5^ from GWAS for RA (Supplementary Table 4). The heterogeneity test showed significant heterogeneity (Q_p value < 0.05, Table 2) in selected IVs of RA on heel BMD and FN-BMD. All tests for MR Egger regression and leave-one-out analysis were negative (p for MR-Egger intercept > 0.05) (Table 2 and Supplementary Figures 6–10), indicating that our MR results were not biased by heterogeneity or horizontal pleiotropy. The value of F statistics for selected IVs is 71.71 (larger than 10), demonstrating that the IVs we selected in this study were powerful enough. As expected, the negative control analyses presented that RA was not associated with myopia, suggesting that the IVs of exposures we selected in this study were appropriate (Supplementary Tables 2, 3).

**Table 2:** Mendelian randomization estimates for RA on BMD.

**Exposure**	**Outcome**	**No. of IVs**	**Heterogeneity tests**		**Directional horizontal pleiotropy test**	**MR results**		
			**Methods**	**Cochran's Q (p)**	**MR-Egger intercept (p)**	**Method**	**Beta**	**P**
**RA**	Heel BMD	4	MR Egger	14.87 (0.001)	3.00e-03 (0.63)	Inverse variance weighted	-2.52	0.20
						MR Egger	-4.33	0.38
			Inverse variance weighted	17.23 (0.001)		Weighted median	-2.96	0.00
						Weighted mode	-3.26	0.04
			MR Egger	5.13 (0.16)	4.00e-02 (0.61)	Inverse variance weighted	3.93	0.80
**RA**	FA-BMD	5				MR Egger	-62.00	0.63
			Inverse variance weighted	5.67 (0.22)		Weighted median	17.68	0.31
						Weighted mode	23.93	0.39
			MR Egger	11.82 (0.02)	8.00e-03 (0.58)	Inverse variance weighted	-4.66	0.50
**RA**	FN-BMD	6				MR Egger	-11.87	0.45
			Inverse variance weighted	12.90 (0.02)		Weighted median	-7.91	0.13
						Weighted mode	-7.78	0.20
			MR Egger	3.53 (0.47)	-6.00e-03 (0.53)	Inverse variance weighted	-4.96	0.32
**RA**	LS-BMD	6				MR Egger	0.49	0.96
			Inverse variance weighted	4.00 (0.55)		Weighted median	-3.20	0.58
						Weighted mode	-3.19	0.66
			MR Egger	4.75 (0.31)	6.34e-05 (0.99)	Inverse variance weighted	-1.74	0.56
**RA**	TB-BMD	6				MR Egger	-1.79	0.77
			Inverse variance weighted	4.75 (0.45)		Weighted median	-2.08	0.53
						Weighted mode	-2.08	0.58

BMD: bone mineral density; RA: rheumatoid arthritis; FA: forearm; FN: femoral neck; LS: lumbar spine; TB: total body; IVs: instrumental variables.

Through IVW analyses, we did not detect any evidence for a causal effect of RA on BMD at any site (beta range from -4.96 to 3.93) (Table 2). The estimates from MR-Egger were consistent with these results (beta range from -62.00 to -2.18). The weighted median (WME) and weighted mode (WMO) analysis detected a significant causal effect of RA on heel BMD (p_WME = 9.93 × 10^-4^, p_WMO = 0.04). When there is absent evidence of directional pleiotropy (p for MR-Egger intercept > 0.05), the IVW method is considered the most reliable indicator in MR analyses [25, 26]. Therefore, we concluded that the causal association between RA and BMD is negative. For the other BMD traits, the results from weighted median and weighted mode analysis were consistent with IVW (Table 2). In summary, combined with the results from different MR methods, we concluded that there is no causal effect of RA on BMD.

## DISCUSSION

In the present study, we used bi-direction Mendelian randomization to figure out whether RA is causally associated with OP, or the other way around. Despite using the largest available public GWAS meta-analyses data, we were unable to demonstrate an association between genetic instruments for RA and BMD, or vice versa. Thus, there was no evidence for a causal relationship between RA and OP according to our MR analyses.

Previous observation studies have shown powerful evidence of an association between active RA and low BMD [27–30]. According to a population-based study, the prevalence of OP had a twofold increase in both male and female RA patients compared with healthy subjects [31]. A Korean cohort including 47,034 RA patients and 235,170 controls also indicated an increased risk of osteoporotic fractures for RA patients across all sexes, age groups, and various anatomic sites, compared with non-RA patients [32]. High disease activity, long disease duration, and joint damage were reported as determinants of reduced BMD in RA patients [15, 33]. Studies on the molecular mechanism also suggested that the pathogenesis of generalized BMD loss and focal erosions had common pathways mediated by osteoclasts, particularly by the receptor activator of nuclear factor kappa B ligand (RANKL) pathway [14, 34, 35].

However, importantly, no conclusions can be drawn on whether RA has a direct influence on OP, or the other way around. Some studies found that focal or generalized bone loss occurred before the diagnosis of RA in some patients, and BMD seems to be predominantly related to demographic factors in those patients without disease-modifying antirheumatic drugs (DMARDs) or corticosteroid treatment [14, 15]. These findings suggested that the reduction of BMD in RA patients might be partially intermediated by other factors, such as antirheumatic treatment. Our MR analysis results found that there is no causal association between RA and OP/BMD, which also suggested a secondary effect of RA on OP/BMD.

Some kinds of classic DMARDs might have effects on the progression of bone formation. For example, methotrexate (MTX) could inhibit the differentiation of osteoblasts and exert direct negative effects on bone metabolism in RA patients [36]. However, Minaur NJ et al. indicated that reduced BMD associated with MTX was due to confounders such as disease activity, and no adverse effect of low-dose MTX on bone formation in RA was detected [37]. Corticosteroids, widely used in treating RA due to their strong suppressive effect on inflammatory activity, can decrease generalized BMD loss. However, as a side-effect, they could also increase BMD loss [38]. Nevertheless, a study including 342 patients with RA found no differences in BMD loss between four common treatment strategies, including high doses of corticosteroids, sequential monotherapy/step-up combination therapy of high doses of MTX, and antitumor necrosis factor-α [15]. Moreover, lack of physical exercise might be another reason for bone loss in patients with RA. Patients with RA usually experience pain, swelling, and immobilization in one or more joints; thus, they are more likely to take less physical activity. Habitual levels of higher impact physical activity were reported to be positively related to lower limb bone strength in older women [39]. A study focused on healthy young men also found that sedentary activities were inversely related to FN-BMD [40].

As far as we know, no MR study on the effect of RA on OP/BMD or OP/BMD on RA has yet been reported. Our study uses several variants summarized from large-scale GWA studies on RA and BMD to date to increase the statistical power to detect causal associations. Compared with the analysis of individual-level data from a small study, a key strength of our research is that effect-size data were drawn from separate large-scale GWASs for exposure and outcome traits so that we can assess the effect sizes more precisely.

However, our study also has certain limitations. First, stratified analyses such as menopausal status would also have been of interest, due to the increased risk of developing OP of postmenopausal women [41], however, since we used summary-level data for two-sample MR analyses, the analyses in specific subgroups were not possible. Otherwise, female sex is also an independent risk factor for RA. However, a Korean large-scale observation study showed an increased risk of osteoporotic fractures for RA patients across all sexes, age groups, and various anatomic sites, suggesting that there might be no sex and age differences in bone loss in patients with RA [32]. Second, compared with other sites, hand BMD is an independent predictor of subsequent radiographic damage since quantitative hand bone loss in RA patients occurs before radiographic joint damage. Therefore, hand BMD may be used as an instrument for the evaluation of bone involvement in RA patients [42]. Unfortunately, we did not obtain available public GWAS data for hand BMD. The forearm BMD summarized data we used in our study included wrist but did not include hand. Nevertheless, some studies have suggested that hand BMD is associated with both the lumbar spine and total hip BMD among postmenopausal women with RA [43], and other RA patients [44]. Thus, using BMD in different sites as phenotypes for OP could reduce the statistical bias in our MR analyses.

In summary, we found no evidence that RA is causally associated with OP/BMD, or the other way around. Therefore, the associations between RA and OP/BMD indicated in previous observational studies are possibly related to secondary effects such as antirheumatic treatment and less physical activity. At present, the clinical treatment of RA is aimed at suppressing inflammatory activity and anti-osteoporosis. Based on our current results, we suggested no indications for anti-osteoporosis treatment in RA patients without other risk factors for OP.

## MATERIALS AND METHODS

### Data sources

Summary statistics for RA were obtained from the MRC IEU OpenGWAS database (https://gwas.mrcieu.ac.uk/), which comprises mainly publicly available GWAS summary data, serving as an input source to a variety of analytical methods, such as Mendelian randomization, fine mapping, and colocalization [45, 46]. The GWAS for RA was examined in imputed genotype data from the UK Biobank study, which included 1,523 cases and 461,487 controls from European populations [45]. RA cases were obtained from the UK Biobank study using Hospital Episode Statistics (HES) with ICD-10 codes M06. Since the absence of the original individual measures, gender- or age-specific analyses were difficult to perform. Fortunately, gender and age were adjusted in the original GWAS analysis. GWAS summary statistics for heel, TB-, FA-, FN- and LS-BMD were obtained from GEFOS (http://www.gefos.org/). The GWAS for heel BMD included 265,627 individuals from the European population in the UK Biobank study. GWAS for heel BMD was estimated from quantitative heel ultrasounds, the age, sex, genotyping array, assessment center, and ancestry informative principal components 1 to 20 were included in the fixed model as covariates [47]. The GWAS dataset for TB-BMD contains summary statistics for a GWAS meta-analysis study involving 66,628 European individuals and was adjusted for age, weight, height, sex, genomic principal components, and other study-specific covariates (such as recruiting center) [23]. GWASs for FA-, FN-, LS-BMD were obtained from a meta-analysis released at GEFOS in 2015 [22]. Separately, 8,143 individuals for FA-BMD, 32,735 individuals for FN-BMD, and 28,498 individuals for LS-BMD from European populations were included, and BMD was adjusted for sex, weight, age, and age squared. More details for assessment, quality control, and association analysis were presented in the original studies [22, 23, 45, 47].

### Selection of genetic variants

In the first stage, genetic variants associated with BMD were used as instrumental SNPs. To satisfy the 3 assumptions for MR analysis, we selected independent SNPs (r^2^ < 0.001) that were strongly (p < 5 × 10^−8^) associated with the exposure. Next, we obtained the association results of the corresponding SNPs with the outcome. If the corresponding SNPs were not available in the outcome data, we used proxy SNPs that were highly correlated (r^2^ > 0.8) with the corresponding SNPs (if possible). To ensure that all corresponding risk factors and outcome alleles were on the same strand, we harmonized the effect of these instrumental SNPs where possible. In the second stage, since there were only 3 SNPs with a p-value less than 5 × 10^−8^ for RA, we broadened the threshold to 1 × 10^-5^ for selecting RA-associated variants as instrumental SNPs. To ensure that the selected IVs have enough power for detecting the causal effect of exposure on the outcome, we calculated the F statistic of selected IVs with an online tool (https://sb452.shinyapps.io/overlap) [48]. The selected IVs with F statistics >10 are considered powerful enough for the causal effect estimate.

### MR analysis

IVW method was conducted as the primary method to estimate the causal effect between exposure and outcome, which was calculated as the effect size of the association between SNP and outcome divided by the effect size of the association between SNP and exposure [49]. When there was no evidence of directional pleiotropy (p for MR-Egger intercept > 0.05) among the selected IVs, the IVW method was considered the most reliable [26]. To ensure the robustness of our results, MR-Egger, weighted median, and weighted mode methods were also performed to estimate the causal effect of exposure on outcome. Detailed information about these MR methods mentioned above can be found in published studies [17, 18]. The MR analyses were performed in the R software (http://www.r-project.org) with the TwoSampleMR package [46]. For the IVs with a p-value < 0.05 for IVW analysis, we then performed MR-PRESSO with the MRPRESSO package [50], which can detect, remove the potential pleiotropic IVs (outliers), and provide the outlier-adjusted estimates. In addition, for pairs of exposure-outcomes with more than 100 IVs, we also performed a recently proposed MR method called Robust Adjusted Profile Score (RAPS) [51], which is unbiased even when there are many (such as hundreds of) weak instruments.

### Sensitivity analysis

To further ensure the robustness of our MR estimates, the following sensitivity analyses were performed: First, Cochran’s Q statistics were employed to assess the heterogeneity among the IVs. Second, MR Egger regression was used to examine whether our MR analyses were driven by the directional horizontal pleiotropy. Moreover, to examine whether the casual association was driven by a single SNP, we performed the leave-one-out analysis.

### Negative control

To further ensure the validity of the selected IVs, we included myopia as a negative control outcome, since no evidence showed that OP or RA is correlated with myopia. The GWAS data for myopia were derived from the FinnGen biobank (https://www.finngen.fi/en), including 621 myopia cases and 93,606 controls from the European population.

## Supplementary Material

Supplementary Figures

Supplementary Table 1

Supplementary Table 2

Supplementary Tables 3 and 4
