# Supplementary material for: Rheumatoid arthritis and osteoporosis: a bi-directional Mendelian randomization study
Source: Aging (Albany NY). 2021 May 18;13(10):14109–30. doi: 10.18632/aging.203029 (PMC8202858; doi:10.18632/aging.203029)
Supplement: Supplementary Figures [file aging-13-203029-s001.pdf]

SUPPLEMENTARY FIGURES

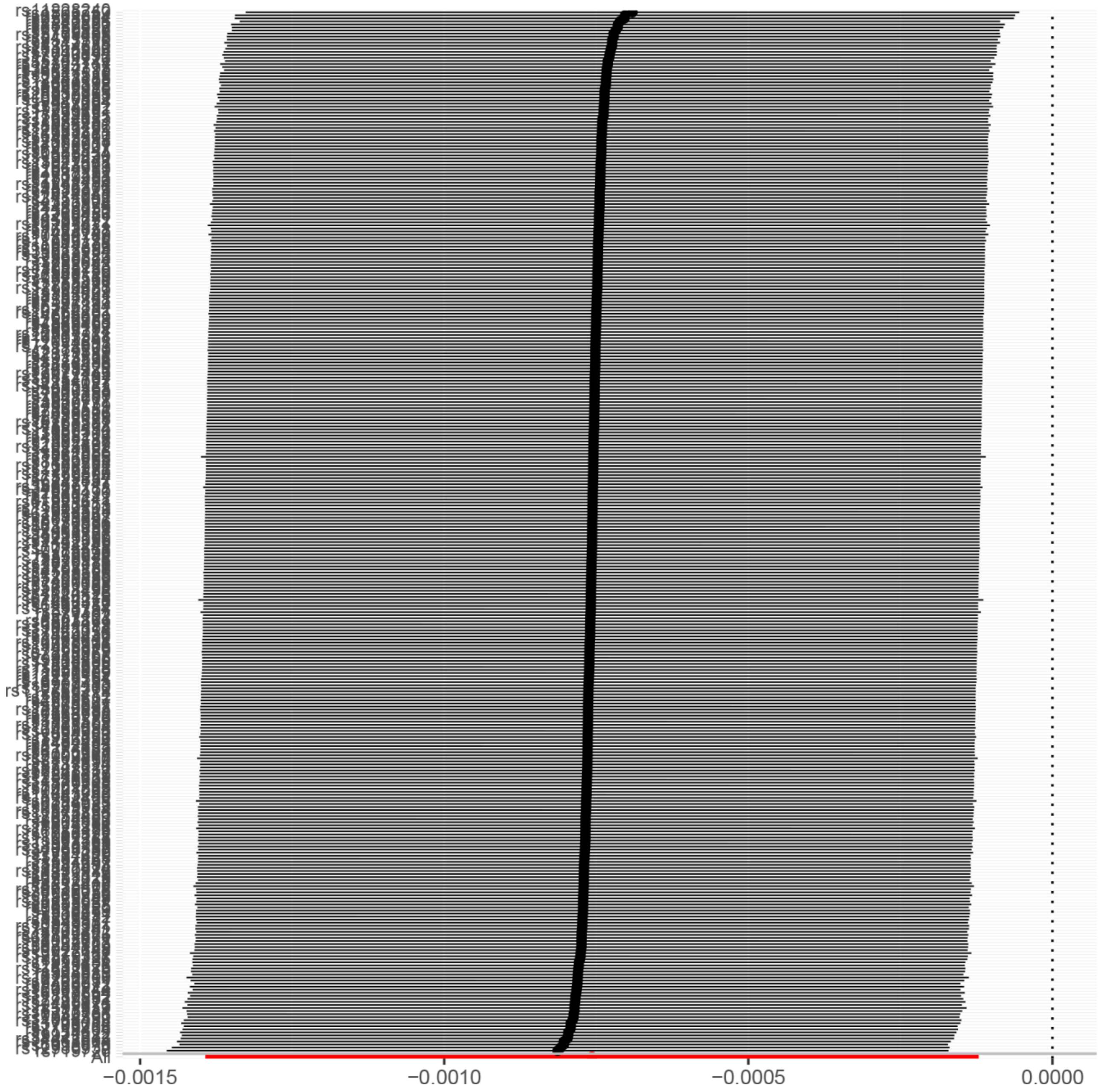

Supplementary Figure 1. MR leave−one−out sensitivity analysis for 'Heel BMD' on 'RA'.

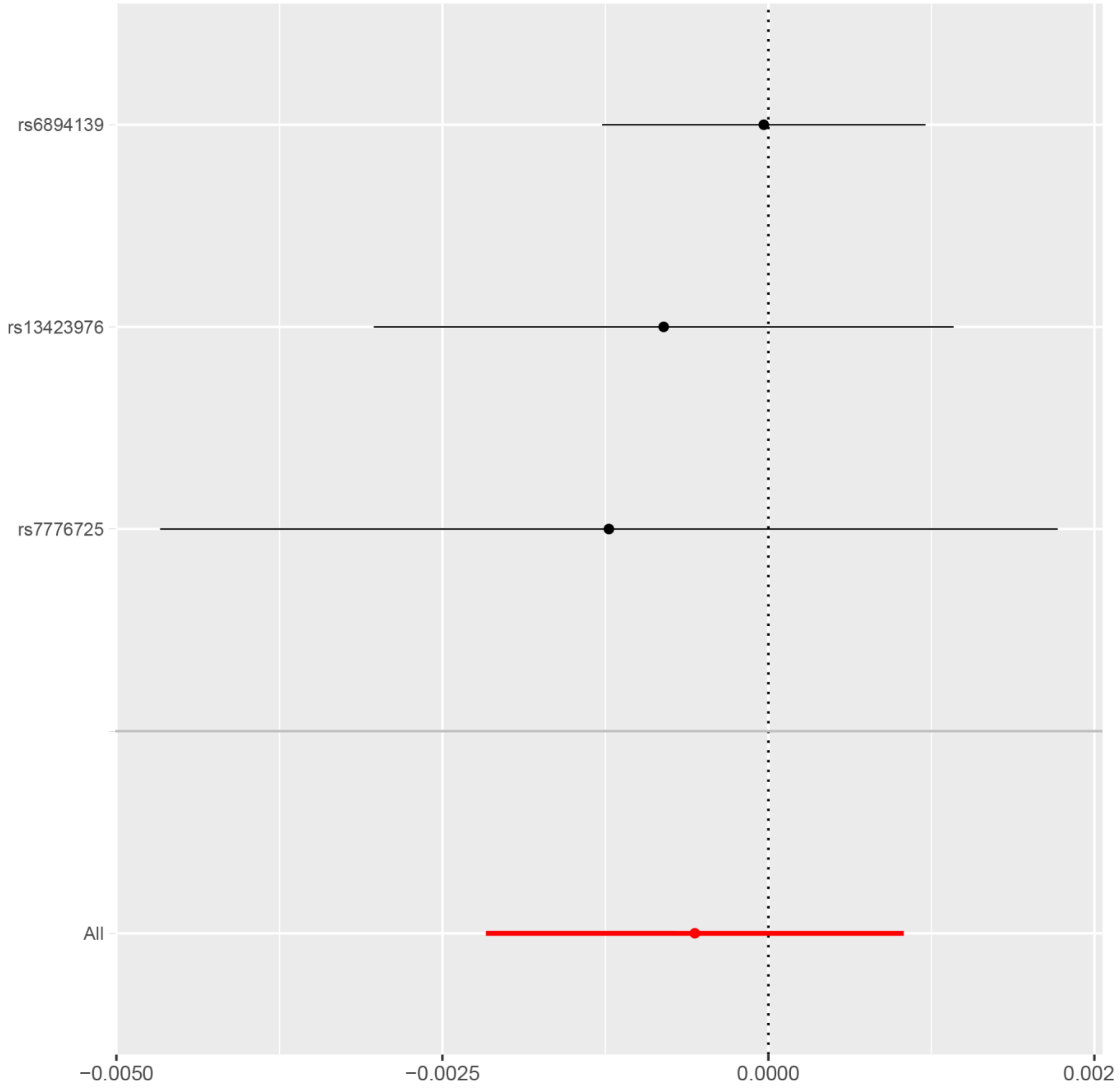

Supplementary Figure 2. MR leave−one−out sensitivity analysis for 'FA-BMD' on 'RA'.

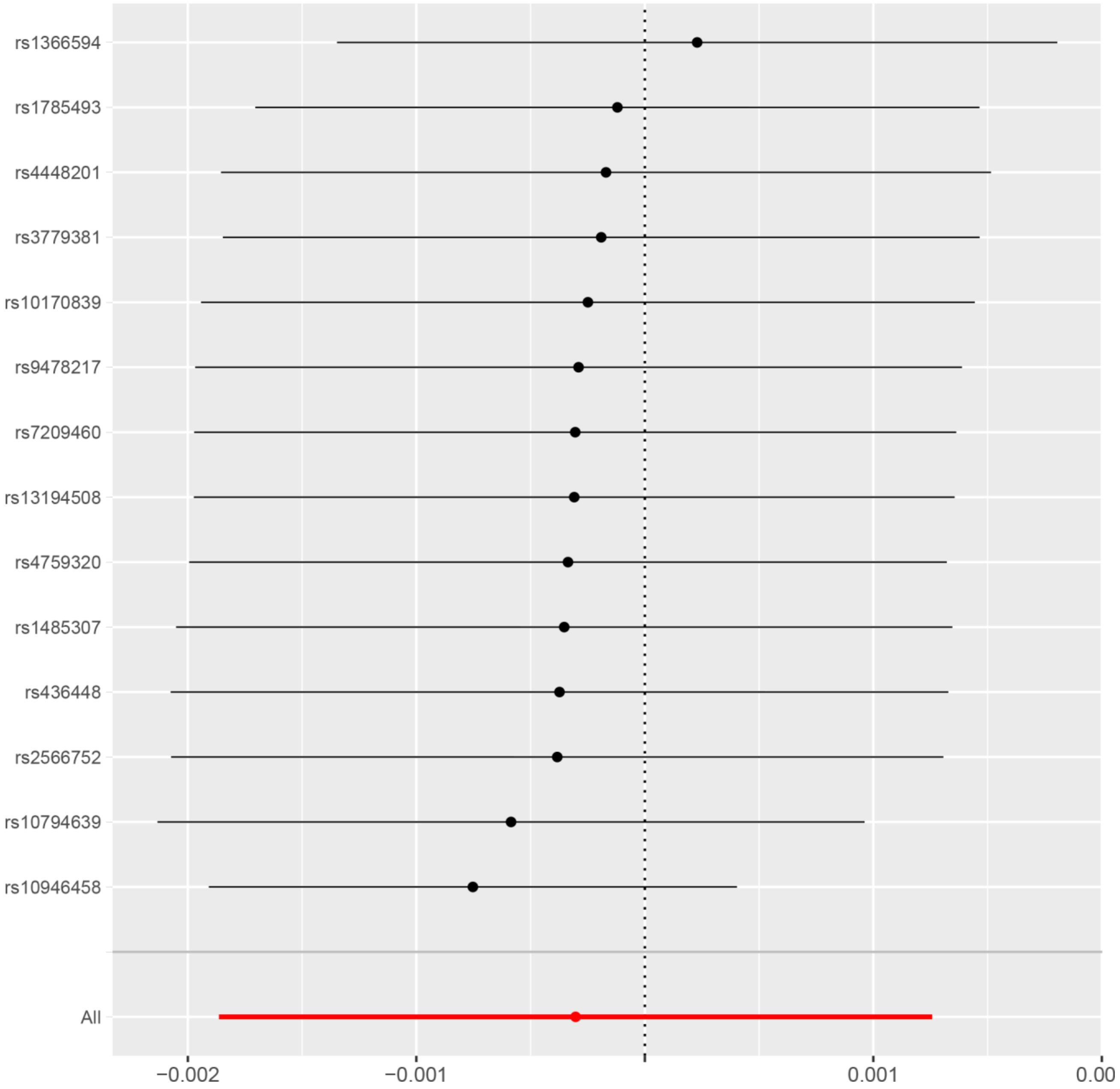

Supplementary Figure 3. MR leave−one−out sensitivity analysis for 'FN-BMD' on 'RA'.

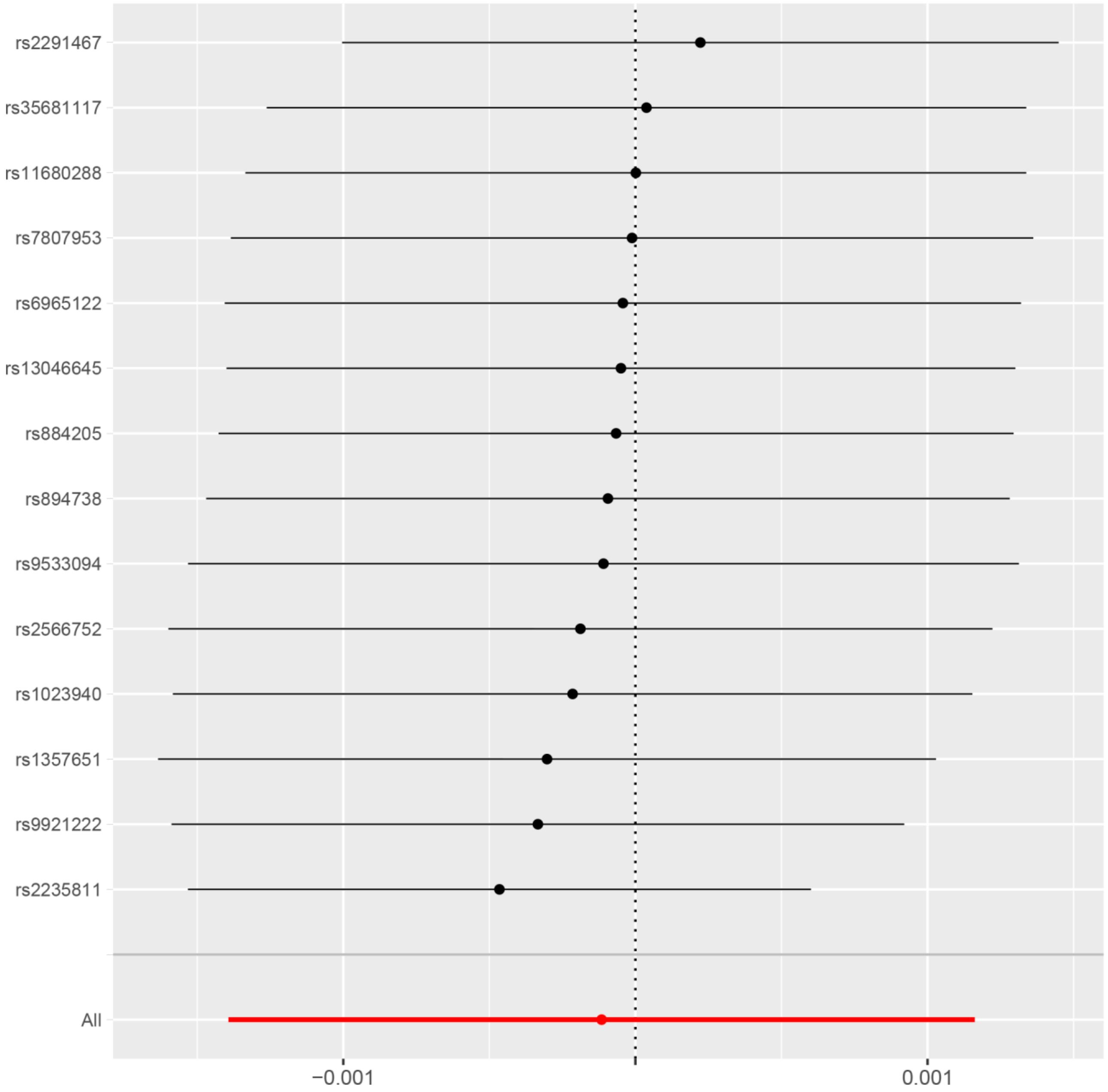

Supplementary Figure 4. MR leave–one–out sensitivity analysis for 'LS-BMD' on 'RA'.

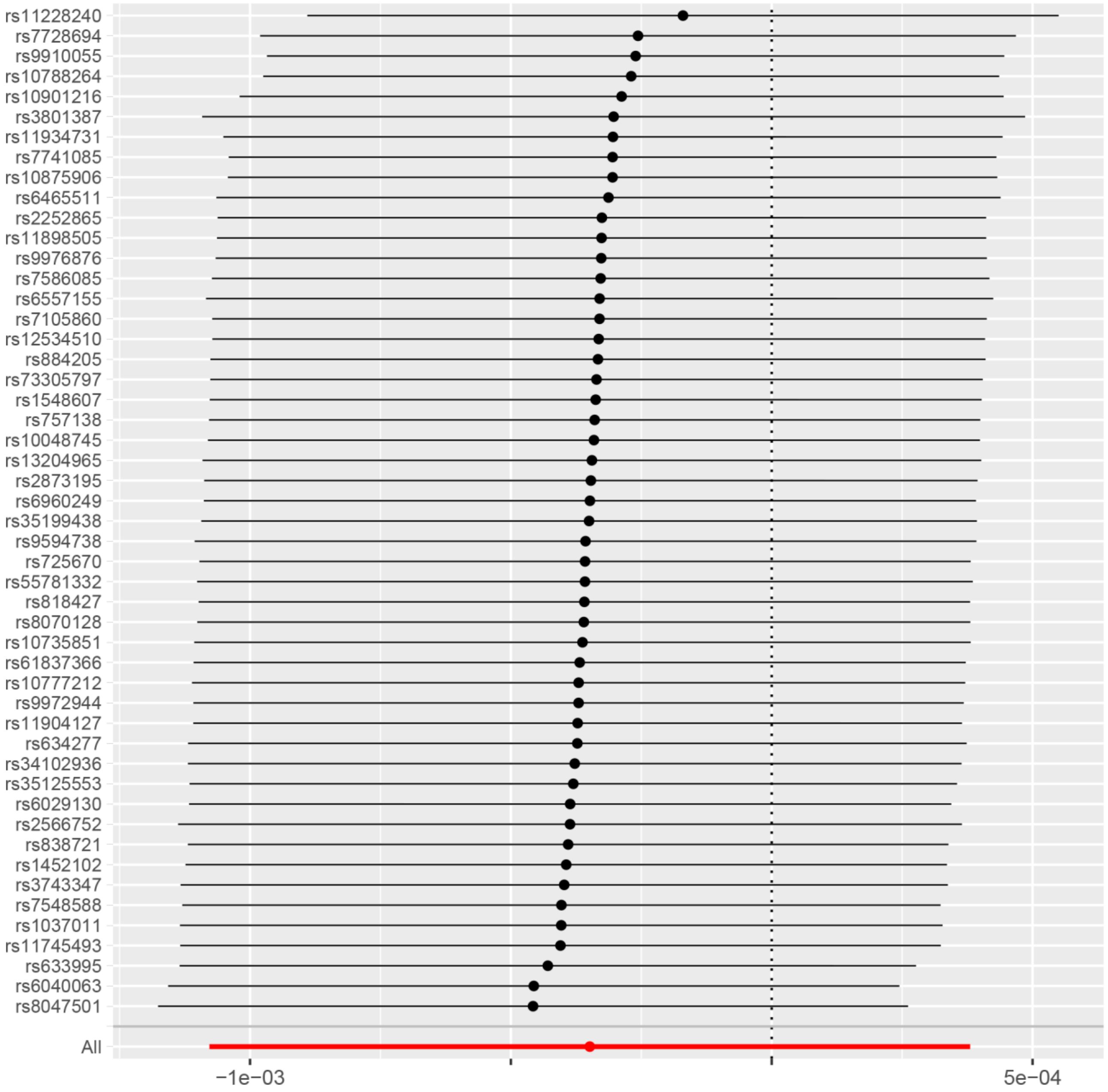

Supplementary Figure 5. MR leave−one−out sensitivity analysis for 'TB-BMD' on 'RA'.

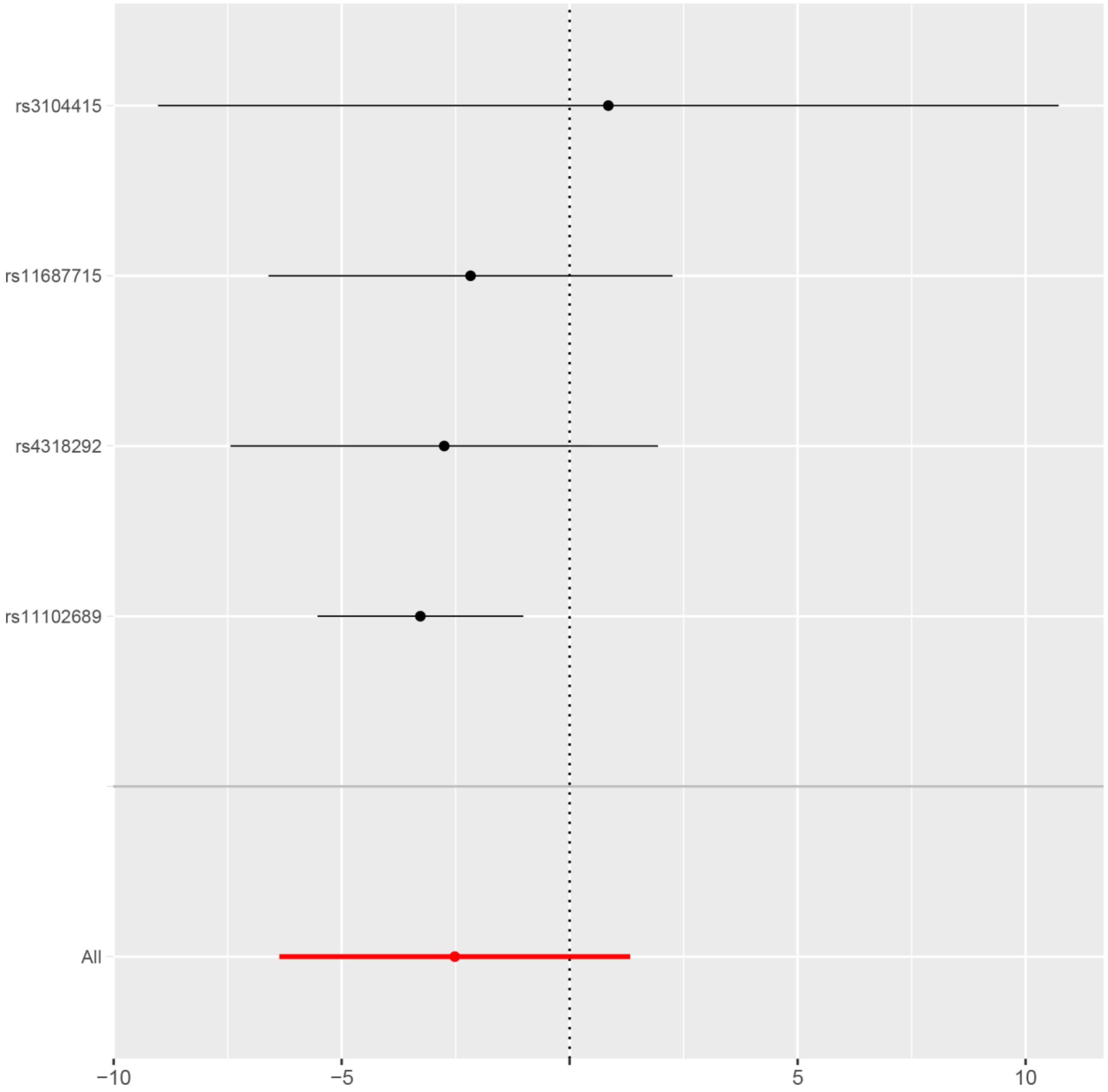

Supplementary Figure 6. MR leave–one–out sensitivity analysis for 'RA' on 'Heel BMD'.

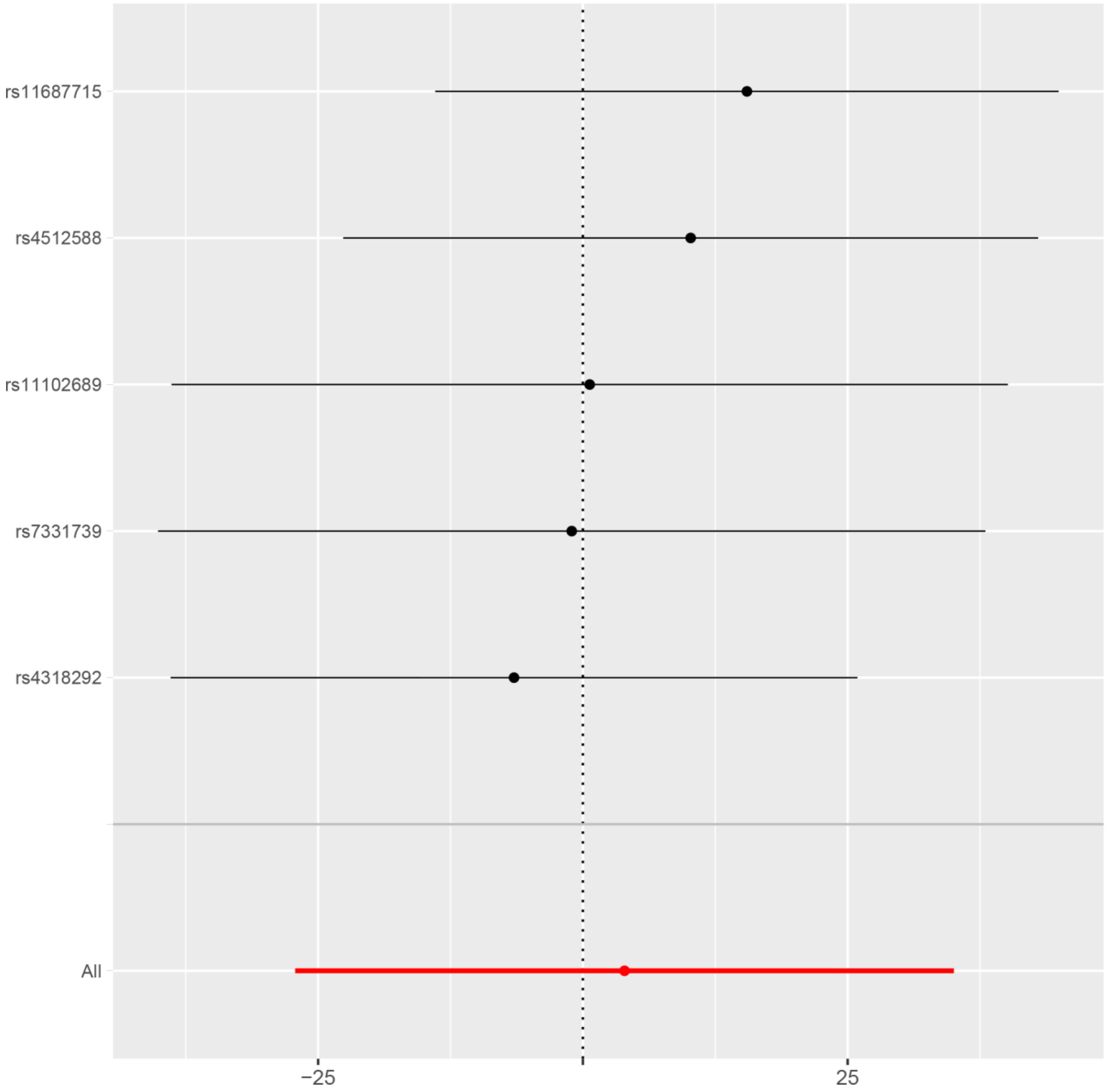

Supplementary Figure 7. MR leave−one−out sensitivity analysis for 'RA' on 'FA-BMD'.

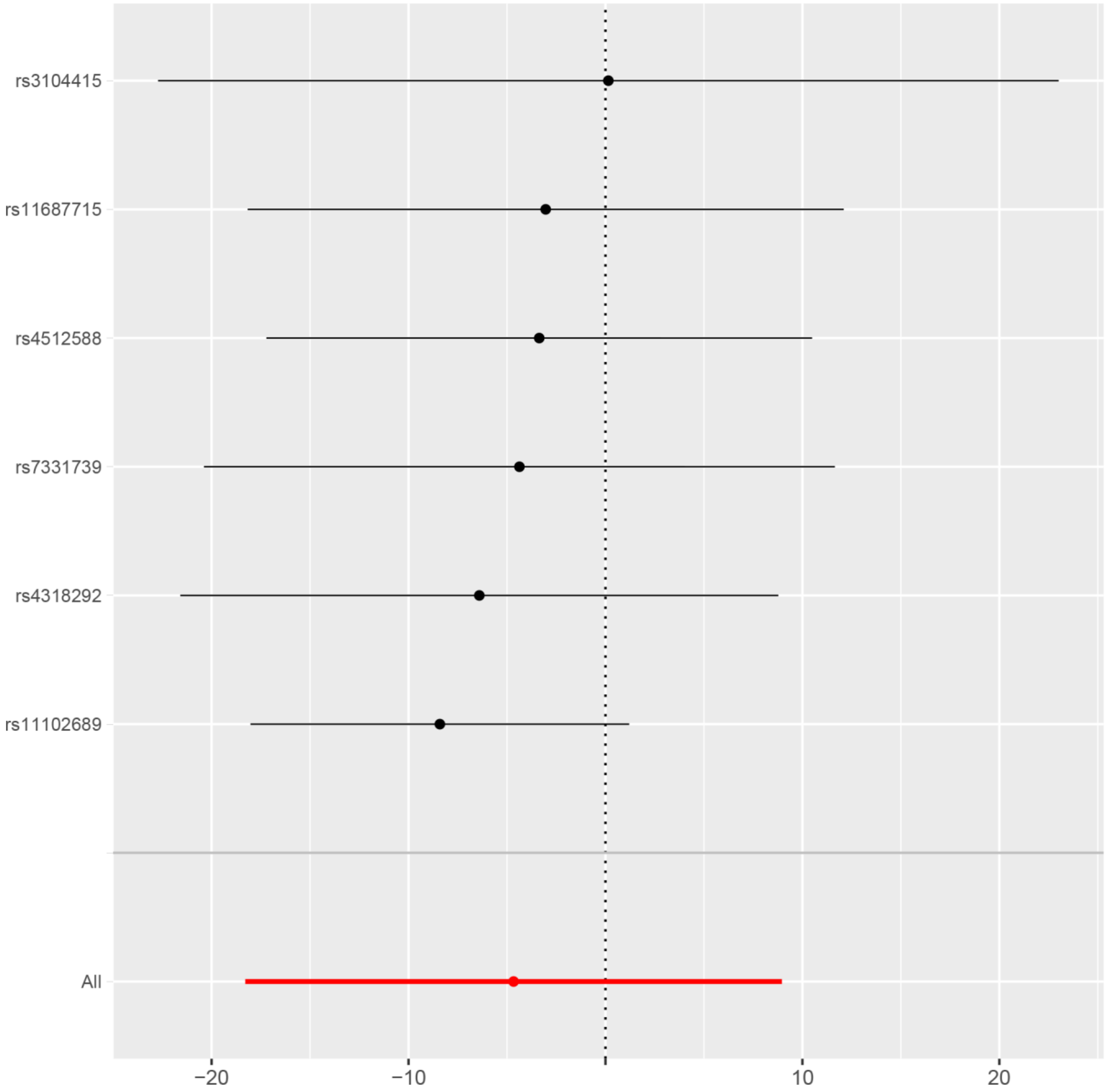

Supplementary Figure 8. MR leave−one−out sensitivity analysis for 'RA' on 'FN-BMD'.

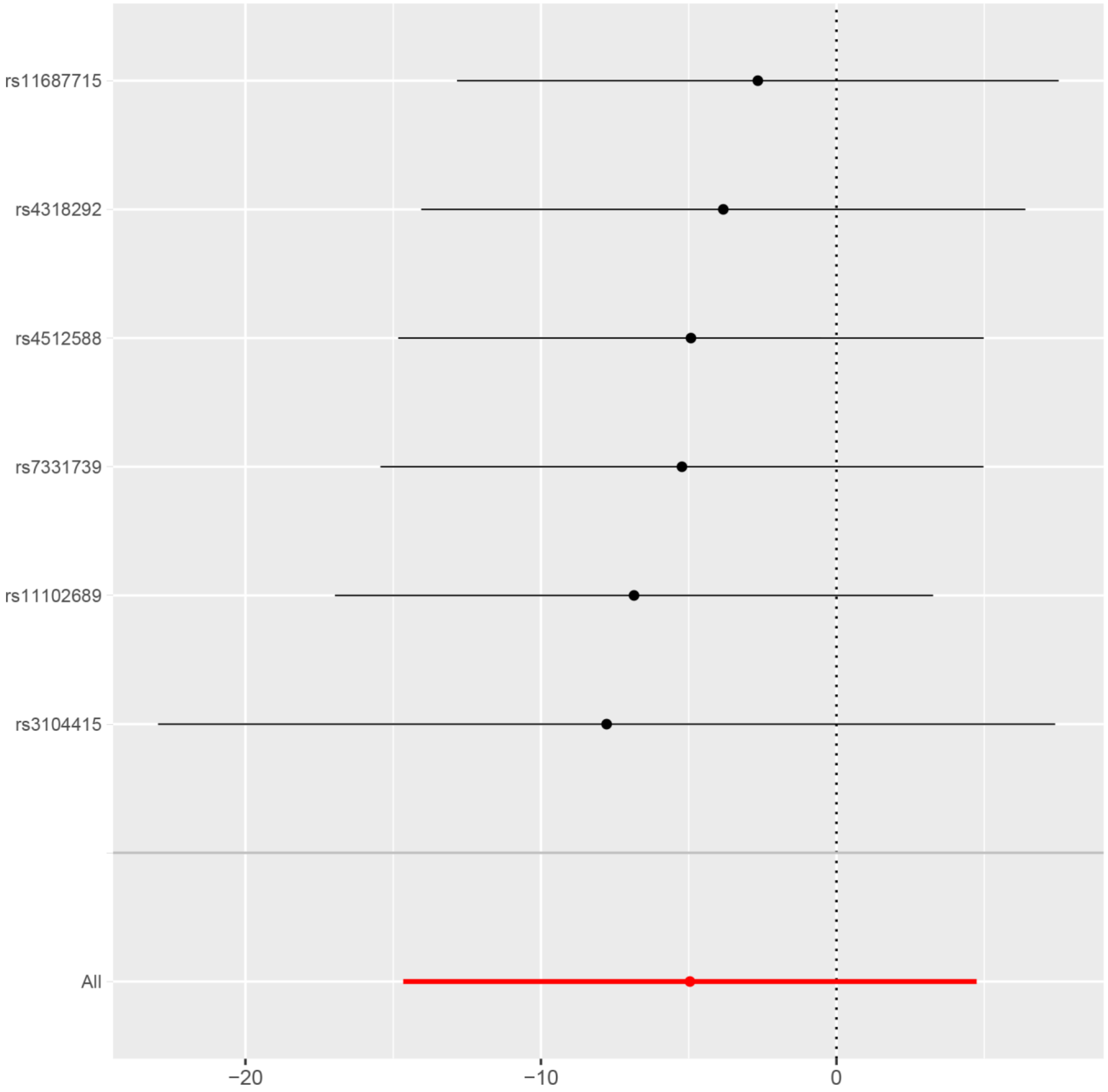

Supplementary Figure 9. MR leave−one−out sensitivity analysis for 'RA' on 'LS-BMD'.

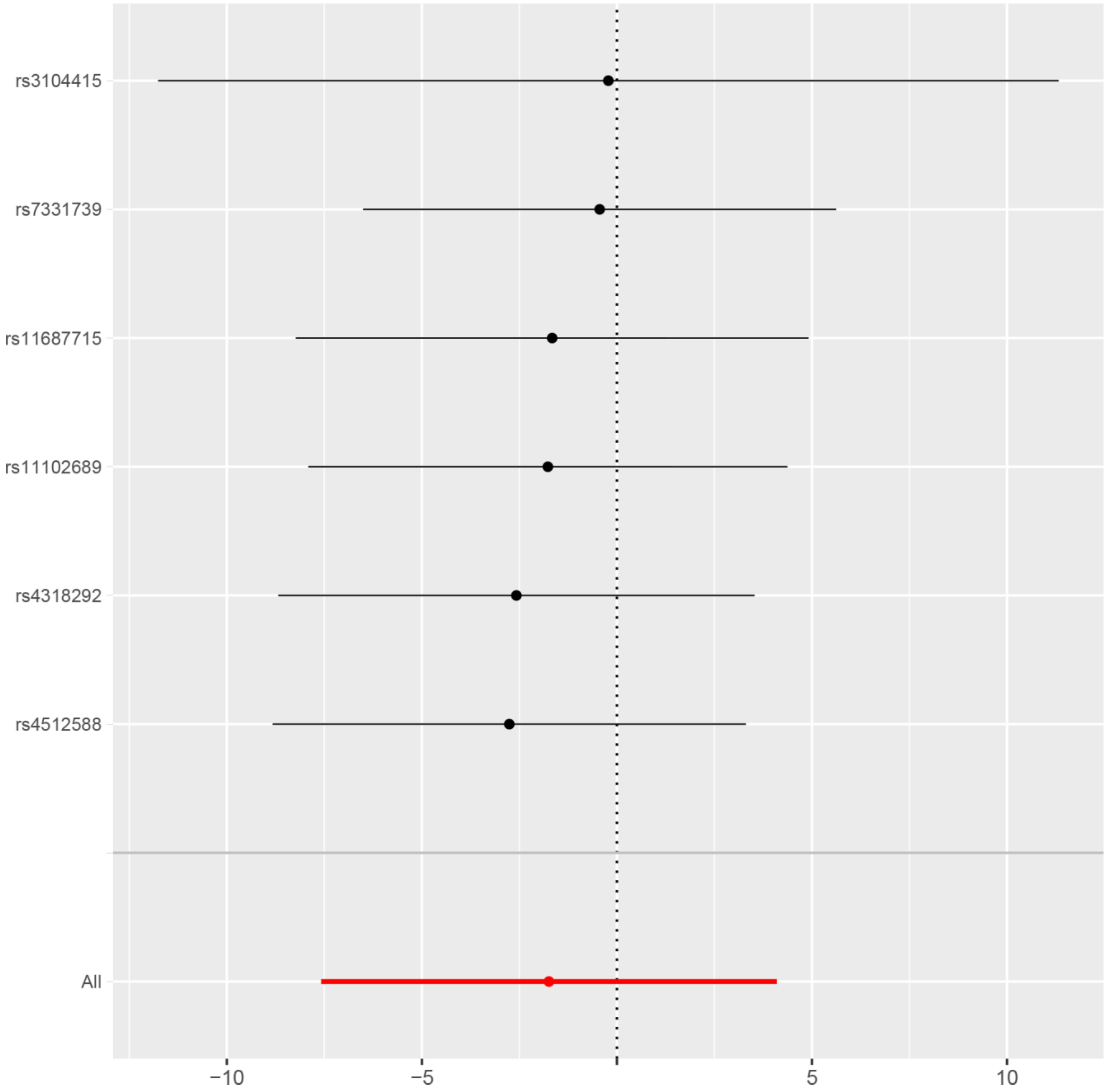

Supplementary Figure 10. MR leave−one−out sensitivity analysis for 'RA' on 'TB-BMD'.
