# Supplementary material for: Rheumatoid arthritis and osteoporosis: a bi-directional Mendelian randomization study
Source: Aging (Albany NY). 2021 May 18;13(10):14109–30. doi: 10.18632/aging.203029 (PMC8202858; doi:10.18632/aging.203029)
Supplement: Supplementary Table 1 [file aging-13-203029-s002.docx]

**Supplementary Table 1. IVs of BMDs for MR analyses in stage 1.**

| Exposure | Outcome | SNP | Effect  allele | Other  allele | GWAS Beta | | GWAS P | |
| --- | --- | --- | --- | --- | --- | --- | --- | --- |
|  |  |  |  |  | **Exposure** | **Outcome** | **Exposure** | **Outcome** |
| Heel BMD | RA | rs10022648 | A | G | 0.016 | 1.59E-04 | 1.80E-18 | 0.2 |
|  |  | rs10063744 | C | G | 0.013 | 3.35E-04 | 4.50E-12 | 0.012 |
|  |  | rs10136356 | G | A | 0.011 | -1.38E-05 | 1.70E-09 | 0.92 |
|  |  | rs10197427 | T | C | 0.057 | -7.48E-05 | 1.00E-200 | 0.55 |
|  |  | rs10279360 | T | C | 0.01 | 8.55E-05 | 9.20E-09 | 0.5 |
|  |  | rs1029830 | C | A | 0.013 | -1.73E-04 | 4.00E-14 | 0.15 |
|  |  | rs10407062 | T | C | 0.014 | 8.67E-05 | 1.90E-14 | 0.47 |
|  |  | rs1043003 | T | C | 0.017 | -1.40E-04 | 1.90E-21 | 0.25 |
|  |  | rs10515269 | C | G | -0.015 | 6.42E-05 | 6.60E-18 | 0.59 |
|  |  | rs1056322 | C | G | 0.013 | 1.26E-04 | 3.10E-12 | 0.32 |
|  |  | rs10750000 | A | G | 0.056 | 2.08E-05 | 1.00E-200 | 0.86 |
|  |  | rs10750766 | C | A | -0.025 | -1.92E-04 | 1.80E-37 | 0.14 |
|  |  | rs10763557 | C | T | 0.019 | -4.22E-05 | 1.30E-24 | 0.72 |
|  |  | rs10764201 | C | T | -0.02 | 6.61E-05 | 1.60E-28 | 0.58 |
|  |  | rs10765568 | C | T | 0.017 | -6.72E-05 | 3.00E-21 | 0.59 |
|  |  | rs10774122 | T | C | 0.013 | -9.51E-05 | 2.30E-12 | 0.44 |
|  |  | rs10779795 | A | G | 0.02 | 2.95E-05 | 3.50E-27 | 0.82 |
|  |  | rs10780789 | C | G | -0.012 | 1.41E-04 | 7.50E-12 | 0.25 |
|  |  | rs10783573 | G | A | 0.016 | -8.61E-06 | 6.00E-17 | 0.95 |
|  |  | rs10788221 | G | A | -0.017 | -2.54E-05 | 1.60E-18 | 0.84 |
|  |  | rs10800531 | A | T | -0.025 | -1.01E-04 | 2.00E-45 | 0.4 |
|  |  | rs10817895 | A | T | -0.016 | 1.31E-04 | 2.50E-13 | 0.33 |
|  |  | rs10842704 | T | G | -0.028 | -5.05E-05 | 2.60E-39 | 0.72 |
|  |  | rs10859561 | C | T | -0.016 | -1.31E-04 | 9.30E-18 | 0.27 |
|  |  | rs10882165 | A | T | -0.013 | -1.25E-04 | 1.40E-11 | 0.3 |
|  |  | rs10885434 | G | C | 0.022 | -1.43E-04 | 1.40E-26 | 0.28 |
|  |  | rs10920352 | T | C | -0.011 | 2.75E-04 | 9.70E-10 | 0.022 |
|  |  | rs10930953 | C | G | 0.019 | -1.89E-04 | 4.20E-24 | 0.13 |
|  |  | rs11003138 | T | C | -0.012 | 1.38E-04 | 1.00E-10 | 0.25 |
|  |  | rs11007241 | G | A | 0.016 | -8.66E-05 | 2.70E-14 | 0.54 |
|  |  | rs11023882 | G | A | 0.013 | -3.88E-05 | 4.70E-12 | 0.75 |
|  |  | rs11048374 | A | C | -0.012 | -3.05E-05 | 2.30E-10 | 0.8 |
|  |  | rs11050203 | A | T | -0.013 | 2.24E-05 | 7.40E-10 | 0.87 |
|  |  | rs11054754 | G | C | 0.014 | 1.26E-04 | 9.50E-15 | 0.29 |
|  |  | rs11067228 | A | G | -0.016 | -9.02E-05 | 1.20E-18 | 0.45 |
|  |  | rs11083780 | T | C | 0.011 | -1.47E-04 | 8.80E-10 | 0.24 |
|  |  | rs11088458 | A | G | 0.044 | 9.76E-05 | 2.80E-105 | 0.46 |
|  |  | rs11100868 | T | C | 0.014 | 3.53E-05 | 7.60E-14 | 0.77 |
|  |  | rs11142400 | G | A | -0.013 | -1.16E-04 | 1.00E-09 | 0.36 |
|  |  | rs11170800 | A | C | -0.011 | 3.55E-05 | 9.80E-09 | 0.77 |
|  |  | rs11175835 | G | A | 0.023 | 6.10E-05 | 6.70E-31 | 0.64 |
|  |  | rs11191149 | C | A | 0.012 | -1.22E-05 | 2.50E-10 | 0.93 |
|  |  | rs11196170 | G | A | 0.03 | -9.31E-05 | 9.40E-42 | 0.51 |
|  |  | rs11228240 | C | T | 0.043 | -3.17E-04 | 6.60E-101 | 0.018 |
|  |  | rs11238754 | C | T | -0.012 | -1.67E-05 | 3.20E-13 | 0.89 |
|  |  | rs11242774 | A | G | 0.014 | -1.56E-04 | 3.30E-14 | 0.2 |
|  |  | rs112766772 | T | C | -0.017 | -6.26E-05 | 3.40E-15 | 0.66 |
|  |  | rs11576308 | G | A | -0.015 | -7.52E-05 | 7.90E-16 | 0.54 |
|  |  | rs11587434 | G | A | -0.012 | 6.17E-05 | 1.80E-09 | 0.65 |
|  |  | rs11622356 | C | T | 0.043 | 8.03E-06 | 2.30E-110 | 0.95 |
|  |  | rs11632673 | A | G | -0.023 | -1.30E-04 | 2.90E-37 | 0.28 |
|  |  | rs11643240 | A | G | 0.015 | 9.81E-05 | 1.50E-13 | 0.47 |
|  |  | rs11668064 | A | G | 0.034 | 2.06E-04 | 1.60E-66 | 0.11 |
|  |  | rs11672782 | G | T | -0.01 | 4.00E-05 | 4.50E-09 | 0.74 |
|  |  | rs11688492 | T | C | 0.013 | 9.58E-06 | 1.30E-13 | 0.94 |
|  |  | rs11696009 | A | C | -0.016 | -3.76E-05 | 1.30E-15 | 0.76 |
|  |  | rs11719201 | C | T | 0.013 | -2.98E-04 | 1.90E-11 | 0.031 |
|  |  | rs11737408 | C | T | 0.011 | 1.02E-04 | 2.80E-08 | 0.45 |
|  |  | rs11880992 | G | A | 0.017 | 2.33E-04 | 3.70E-20 | 0.054 |
|  |  | rs11894900 | C | G | -0.015 | -7.04E-05 | 3.00E-14 | 0.59 |
|  |  | rs11934731 | G | A | 0.036 | -1.06E-04 | 6.10E-74 | 0.41 |
|  |  | rs11935978 | A | G | 0.011 | 5.70E-05 | 7.80E-09 | 0.63 |
|  |  | rs12077443 | C | T | -0.01 | 7.50E-05 | 8.30E-10 | 0.53 |
|  |  | rs12121554 | G | A | -0.024 | -1.96E-05 | 5.50E-34 | 0.88 |
|  |  | rs12149673 | A | G | -0.029 | -1.51E-04 | 4.30E-47 | 0.24 |
|  |  | rs12210993 | G | A | 0.022 | 2.64E-05 | 1.20E-35 | 0.82 |
|  |  | rs12276167 | T | G | 0.014 | 4.64E-05 | 2.50E-13 | 0.7 |
|  |  | rs12323717 | A | G | -0.015 | 2.88E-04 | 4.00E-14 | 0.021 |
|  |  | rs12346689 | C | T | 0.019 | 5.79E-05 | 1.90E-23 | 0.64 |
|  |  | rs12372718 | A | G | 0.015 | -1.70E-04 | 3.90E-16 | 0.16 |
|  |  | rs12414040 | T | C | -0.011 | 1.84E-04 | 3.80E-08 | 0.12 |
|  |  | rs12443188 | T | A | -0.022 | 2.30E-04 | 3.40E-25 | 0.11 |
|  |  | rs1255471 | A | C | -0.01 | 1.35E-04 | 2.20E-08 | 0.26 |
|  |  | rs1256065 | G | T | -0.012 | 3.63E-05 | 3.50E-11 | 0.76 |
|  |  | rs12587727 | C | T | -0.011 | 1.13E-04 | 2.40E-08 | 0.41 |
|  |  | rs12614693 | G | A | 0.014 | -9.03E-05 | 7.50E-12 | 0.51 |
|  |  | rs12683791 | T | C | -0.029 | 1.27E-04 | 1.60E-46 | 0.34 |
|  |  | rs12703135 | A | G | 0.012 | 5.72E-06 | 1.10E-09 | 0.97 |
|  |  | rs12733821 | G | C | -0.016 | 1.75E-04 | 1.40E-17 | 0.16 |
|  |  | rs12811685 | C | G | 0.018 | 1.45E-04 | 9.10E-20 | 0.25 |
|  |  | rs12932885 | C | T | 0.024 | -1.60E-04 | 2.90E-39 | 0.18 |
|  |  | rs12973365 | A | G | -0.01 | -1.56E-04 | 4.40E-08 | 0.2 |
|  |  | rs12974515 | A | G | 0.01 | -1.93E-05 | 1.60E-08 | 0.87 |
|  |  | rs12986939 | G | A | 0.05 | 1.49E-04 | 1.20E-140 | 0.26 |
|  |  | rs13002567 | T | C | -0.018 | -5.46E-05 | 6.20E-22 | 0.67 |
|  |  | rs13022378 | C | T | -0.023 | 1.13E-04 | 1.30E-34 | 0.35 |
|  |  | rs13065094 | G | A | -0.021 | -3.11E-05 | 1.20E-34 | 0.8 |
|  |  | rs13070996 | G | T | 0.014 | 3.97E-05 | 1.40E-13 | 0.77 |
|  |  | rs13083728 | G | A | -0.017 | 1.26E-04 | 1.90E-14 | 0.36 |
|  |  | rs13088318 | A | G | -0.015 | 2.43E-04 | 8.20E-15 | 0.054 |
|  |  | rs13154707 | A | G | -0.021 | 5.86E-05 | 1.10E-23 | 0.67 |
|  |  | rs13179493 | T | C | -0.03 | 2.41E-04 | 5.20E-51 | 0.07 |
|  |  | rs13230922 | C | T | 0.015 | 1.47E-04 | 6.30E-15 | 0.26 |
|  |  | rs13379337 | C | A | -0.022 | -1.09E-04 | 1.10E-31 | 0.36 |
|  |  | rs13427681 | G | C | 0.011 | -7.95E-05 | 1.50E-09 | 0.51 |
|  |  | rs134622 | T | G | 0.04 | 1.36E-04 | 6.20E-95 | 0.29 |
|  |  | rs1392788 | A | C | 0.011 | -4.37E-05 | 4.00E-09 | 0.72 |
|  |  | rs1426889 | C | T | -0.012 | -9.44E-06 | 1.70E-10 | 0.94 |
|  |  | rs1444436 | C | G | 0.016 | 2.10E-05 | 4.80E-17 | 0.87 |
|  |  | rs1455420 | T | G | -0.01 | 8.62E-05 | 9.30E-09 | 0.48 |
|  |  | rs1455816 | A | G | 0.012 | 5.10E-05 | 4.70E-10 | 0.69 |
|  |  | rs1463597 | A | G | -0.012 | 1.93E-04 | 4.50E-12 | 0.11 |
|  |  | rs1468316 | T | A | -0.01 | -2.52E-05 | 8.60E-09 | 0.84 |
|  |  | rs1484434 | G | T | 0.012 | 9.97E-05 | 8.50E-10 | 0.45 |
|  |  | rs1500920 | T | C | 0.016 | -1.28E-04 | 2.40E-17 | 0.3 |
|  |  | rs1502201 | G | A | 0.017 | -4.45E-05 | 7.80E-19 | 0.75 |
|  |  | rs1533142 | C | G | 0.023 | -2.71E-05 | 7.90E-28 | 0.85 |
|  |  | rs1535571 | A | G | -0.013 | -1.71E-04 | 1.50E-12 | 0.15 |
|  |  | rs1548607 | A | G | 0.018 | -5.19E-05 | 7.60E-23 | 0.68 |
|  |  | rs1550270 | T | C | -0.016 | -5.98E-05 | 2.20E-15 | 0.65 |
|  |  | rs1555958 | C | G | -0.031 | -5.78E-05 | 1.80E-53 | 0.65 |
|  |  | rs1564983 | C | T | -0.022 | 1.20E-04 | 1.50E-30 | 0.34 |
|  |  | rs1622638 | G | A | -0.015 | 1.18E-04 | 3.60E-16 | 0.33 |
|  |  | rs167365 | C | G | 0.019 | 7.56E-05 | 3.30E-23 | 0.54 |
|  |  | rs16884419 | G | A | 0.014 | 3.19E-05 | 3.70E-11 | 0.82 |
|  |  | rs1706708 | G | A | 0.029 | 1.82E-04 | 2.90E-54 | 0.15 |
|  |  | rs174598 | G | A | -0.014 | 7.66E-05 | 9.60E-13 | 0.55 |
|  |  | rs17684825 | T | G | -0.012 | 2.35E-04 | 9.70E-11 | 0.051 |
|  |  | rs17700633 | G | A | -0.013 | 1.24E-04 | 2.80E-10 | 0.34 |
|  |  | rs1777277 | C | A | 0.011 | -3.61E-05 | 2.40E-09 | 0.76 |
|  |  | rs17796692 | C | G | 0.018 | -2.87E-04 | 6.10E-19 | 0.026 |
|  |  | rs178896 | T | A | 0.016 | -7.59E-06 | 4.00E-15 | 0.96 |
|  |  | rs1844776 | T | C | -0.012 | 1.34E-04 | 2.60E-11 | 0.27 |
|  |  | rs1861000 | T | C | -0.062 | 5.20E-05 | 1.00E-200 | 0.66 |
|  |  | rs1865712 | T | C | -0.012 | -7.79E-05 | 1.80E-09 | 0.55 |
|  |  | rs1897465 | A | G | 0.013 | 1.67E-04 | 5.10E-09 | 0.2 |
|  |  | rs1991431 | G | A | 0.018 | 1.66E-04 | 3.70E-23 | 0.17 |
|  |  | rs1999536 | G | C | -0.021 | 8.80E-05 | 3.00E-29 | 0.47 |
|  |  | rs2005617 | T | C | -0.013 | -2.01E-04 | 2.20E-12 | 0.1 |
|  |  | rs2052480 | G | A | -0.027 | -4.96E-05 | 1.50E-41 | 0.71 |
|  |  | rs206432 | G | T | -0.016 | -1.32E-04 | 4.20E-18 | 0.27 |
|  |  | rs2069442 | G | C | 0.019 | 1.96E-05 | 7.80E-21 | 0.89 |
|  |  | rs2085490 | A | G | -0.013 | 2.30E-05 | 3.80E-10 | 0.85 |
|  |  | rs210374 | A | T | -0.014 | 1.77E-04 | 8.70E-12 | 0.19 |
|  |  | rs212417 | G | A | 0.029 | 5.56E-05 | 2.60E-53 | 0.66 |
|  |  | rs215226 | A | G | -0.024 | -1.08E-04 | 2.10E-40 | 0.38 |
|  |  | rs2174633 | A | C | -0.016 | -7.66E-05 | 1.10E-14 | 0.57 |
|  |  | rs2179519 | T | A | 0.011 | -1.10E-04 | 2.50E-09 | 0.42 |
|  |  | rs2239626 | T | C | -0.017 | -2.14E-04 | 4.00E-17 | 0.099 |
|  |  | rs2240226 | C | T | -0.034 | 2.62E-04 | 2.50E-62 | 0.054 |
|  |  | rs2254027 | C | A | -0.019 | 5.50E-05 | 1.60E-20 | 0.67 |
|  |  | rs2256835 | G | C | -0.012 | -1.17E-04 | 3.90E-08 | 0.37 |
|  |  | rs2302407 | G | A | 0.016 | -3.60E-05 | 4.20E-18 | 0.77 |
|  |  | rs2306272 | T | C | -0.012 | 4.07E-05 | 1.90E-09 | 0.76 |
|  |  | rs2337106 | C | G | -0.019 | 8.02E-06 | 2.30E-26 | 0.95 |
|  |  | rs2353551 | A | G | -0.011 | 6.53E-05 | 5.10E-09 | 0.61 |
|  |  | rs2374653 | T | G | 0.012 | -2.13E-04 | 3.30E-11 | 0.075 |
|  |  | rs2382801 | C | A | -0.02 | 7.38E-05 | 7.90E-26 | 0.54 |
|  |  | rs2388792 | A | G | -0.013 | -6.51E-05 | 3.20E-12 | 0.59 |
|  |  | rs241770 | G | A | -0.012 | 3.93E-04 | 9.60E-10 | 0.002 |
|  |  | rs2430689 | C | G | 0.016 | -4.66E-05 | 1.80E-18 | 0.7 |
|  |  | rs2442599 | G | A | -0.015 | 1.40E-05 | 4.20E-13 | 0.92 |
|  |  | rs2491105 | T | C | -0.023 | 3.18E-04 | 4.80E-25 | 0.025 |
|  |  | rs2509353 | C | T | 0.021 | -1.13E-04 | 6.90E-30 | 0.35 |
|  |  | rs2546985 | A | G | 0.015 | -1.03E-04 | 6.50E-12 | 0.46 |
|  |  | rs2553772 | T | G | -0.028 | 8.14E-05 | 3.20E-57 | 0.5 |
|  |  | rs2566752 | T | C | -0.036 | -3.55E-05 | 1.30E-78 | 0.77 |
|  |  | rs258223 | T | C | 0.011 | -1.46E-04 | 1.10E-08 | 0.25 |
|  |  | rs2639953 | G | A | 0.028 | 2.83E-05 | 2.50E-57 | 0.81 |
|  |  | rs264647 | C | T | -0.02 | -3.32E-05 | 8.10E-30 | 0.78 |
|  |  | rs2722176 | T | C | -0.013 | -1.11E-06 | 3.80E-12 | 0.99 |
|  |  | rs2737252 | G | A | -0.035 | -1.16E-04 | 9.80E-67 | 0.38 |
|  |  | rs2761884 | G | T | 0.05 | -1.94E-04 | 3.10E-164 | 0.11 |
|  |  | rs277402 | G | A | -0.013 | -2.86E-05 | 1.40E-09 | 0.84 |
|  |  | rs2830913 | G | A | -0.024 | -1.38E-04 | 8.80E-37 | 0.25 |
|  |  | rs28364580 | G | A | 0.022 | -2.96E-04 | 2.20E-25 | 0.033 |
|  |  | rs28557305 | G | A | 0.026 | -1.47E-04 | 1.10E-42 | 0.24 |
|  |  | rs2929308 | T | A | 0.044 | 2.31E-04 | 1.80E-132 | 0.053 |
|  |  | rs2944590 | G | A | -0.02 | -2.10E-04 | 2.90E-28 | 0.079 |
|  |  | rs2982571 | A | T | -0.077 | -9.37E-05 | 1.00E-200 | 0.44 |
|  |  | rs3118906 | G | A | -0.026 | 2.28E-04 | 3.50E-38 | 0.085 |
|  |  | rs3131593 | G | C | 0.014 | 1.73E-04 | 8.50E-10 | 0.21 |
|  |  | rs314261 | A | C | -0.013 | -1.09E-04 | 2.40E-12 | 0.36 |
|  |  | rs330091 | A | G | -0.019 | -1.45E-04 | 2.00E-16 | 0.3 |
|  |  | rs34068557 | C | T | 0.012 | 1.46E-04 | 1.20E-09 | 0.23 |
|  |  | rs344035 | G | A | 0.015 | 2.23E-04 | 6.00E-15 | 0.061 |
|  |  | rs34435227 | A | G | 0.013 | -6.10E-05 | 2.70E-14 | 0.61 |
|  |  | rs34441013 | G | A | -0.032 | -2.09E-04 | 1.30E-55 | 0.12 |
|  |  | rs34627117 | C | G | -0.022 | 2.12E-04 | 2.00E-29 | 0.11 |
|  |  | rs34853396 | G | T | 0.012 | -1.23E-05 | 6.10E-11 | 0.92 |
|  |  | rs34879158 | A | C | -0.017 | 2.83E-05 | 1.20E-15 | 0.83 |
|  |  | rs35155027 | G | C | 0.016 | 1.30E-04 | 1.10E-17 | 0.29 |
|  |  | rs35657711 | T | C | -0.051 | 4.41E-05 | 4.90E-116 | 0.75 |
|  |  | rs35713954 | G | T | 0.011 | -1.44E-04 | 4.70E-09 | 0.23 |
|  |  | rs359942 | C | T | -0.01 | 1.45E-06 | 1.20E-08 | 0.99 |
|  |  | rs36016056 | G | C | -0.019 | -1.30E-04 | 1.70E-19 | 0.34 |
|  |  | rs36124395 | G | A | 0.01 | -3.76E-04 | 1.60E-08 | 0.002 |
|  |  | rs368510 | G | A | -0.024 | -1.42E-04 | 1.10E-34 | 0.26 |
|  |  | rs370387 | G | A | -0.047 | 2.86E-05 | 2.80E-149 | 0.81 |
|  |  | rs3747010 | G | A | -0.012 | -1.05E-04 | 8.50E-09 | 0.43 |
|  |  | rs3751908 | A | G | -0.019 | 1.19E-04 | 1.20E-22 | 0.33 |
|  |  | rs3752075 | T | C | -0.01 | -2.01E-04 | 2.20E-08 | 0.098 |
|  |  | rs3760456 | C | T | 0.023 | -9.29E-05 | 1.50E-36 | 0.44 |
|  |  | rs3765971 | C | T | -0.028 | 8.56E-05 | 5.00E-53 | 0.49 |
|  |  | rs3777787 | C | A | 0.052 | 1.29E-05 | 3.70E-182 | 0.91 |
|  |  | rs3796014 | A | G | -0.051 | -8.54E-05 | 5.70E-146 | 0.52 |
|  |  | rs3828559 | A | G | 0.015 | 8.01E-05 | 5.70E-12 | 0.56 |
|  |  | rs3829849 | C | T | -0.014 | -1.56E-04 | 2.00E-14 | 0.21 |
|  |  | rs3848474 | G | A | -0.015 | -1.19E-04 | 1.30E-16 | 0.32 |
|  |  | rs3936185 | T | C | 0.02 | 7.49E-05 | 7.10E-25 | 0.55 |
|  |  | rs3941888 | G | A | -0.013 | -3.27E-05 | 7.50E-12 | 0.8 |
|  |  | rs4358110 | C | A | -0.014 | 4.58E-05 | 1.00E-11 | 0.73 |
|  |  | rs4360494 | G | C | -0.019 | 2.33E-05 | 4.10E-25 | 0.85 |
|  |  | rs4383904 | T | C | -0.045 | 7.88E-05 | 3.80E-132 | 0.53 |
|  |  | rs4401555 | T | A | 0.011 | 5.25E-05 | 6.90E-10 | 0.66 |
|  |  | rs4450871 | A | G | -0.011 | 1.61E-04 | 5.70E-11 | 0.18 |
|  |  | rs4488059 | T | C | 0.017 | -1.26E-04 | 5.30E-18 | 0.31 |
|  |  | rs4505759 | C | T | -0.057 | 2.07E-04 | 5.80E-193 | 0.11 |
|  |  | rs4595506 | A | G | 0.023 | -1.01E-05 | 6.20E-37 | 0.93 |
|  |  | rs4600907 | T | C | -0.015 | 1.84E-04 | 3.60E-15 | 0.14 |
|  |  | rs4635400 | G | A | 0.05 | 4.99E-06 | 1.00E-155 | 0.97 |
|  |  | rs4693374 | A | C | -0.019 | 6.35E-05 | 8.20E-27 | 0.59 |
|  |  | rs4694691 | G | A | -0.009 | 1.21E-04 | 3.70E-08 | 0.31 |
|  |  | rs4724126 | G | T | -0.017 | -7.02E-06 | 4.20E-18 | 0.96 |
|  |  | rs4739697 | A | G | 0.019 | 4.15E-05 | 2.40E-24 | 0.74 |
|  |  | rs4782351 | A | G | 0.015 | -1.55E-04 | 2.70E-16 | 0.21 |
|  |  | rs4806862 | G | T | -0.02 | 4.42E-05 | 1.10E-24 | 0.73 |
|  |  | rs4810131 | C | G | 0.01 | -6.93E-05 | 4.90E-08 | 0.57 |
|  |  | rs4836373 | T | C | 0.019 | -1.12E-04 | 1.40E-25 | 0.35 |
|  |  | rs4876361 | G | A | 0.013 | 8.24E-05 | 1.30E-09 | 0.54 |
|  |  | rs4884522 | C | A | 0.011 | -4.11E-05 | 2.70E-10 | 0.73 |
|  |  | rs4886486 | G | C | -0.012 | 9.15E-05 | 1.90E-08 | 0.5 |
|  |  | rs4889490 | G | T | -0.012 | 9.53E-05 | 4.30E-11 | 0.43 |
|  |  | rs4915526 | C | T | 0.016 | -7.04E-05 | 5.00E-16 | 0.58 |
|  |  | rs4960293 | T | G | 0.014 | -5.65E-05 | 1.50E-15 | 0.64 |
|  |  | rs55704141 | G | A | -0.029 | 1.60E-04 | 1.20E-51 | 0.19 |
|  |  | rs55709850 | G | T | 0.017 | 1.06E-06 | 2.60E-17 | 0.99 |
|  |  | rs55771168 | T | C | 0.011 | 1.76E-05 | 2.10E-08 | 0.9 |
|  |  | rs55888531 | C | T | 0.021 | 4.71E-05 | 6.30E-29 | 0.71 |
|  |  | rs56371096 | C | T | -0.009 | 1.23E-05 | 3.50E-08 | 0.92 |
|  |  | rs56744189 | C | A | 0.018 | 1.68E-04 | 5.70E-20 | 0.21 |
|  |  | rs571356 | G | A | 0.016 | -3.73E-05 | 6.30E-15 | 0.77 |
|  |  | rs5762768 | A | G | 0.02 | 5.07E-05 | 2.50E-26 | 0.67 |
|  |  | rs594647 | C | T | -0.01 | -1.79E-04 | 3.10E-09 | 0.14 |
|  |  | rs597319 | A | G | 0.066 | 2.36E-05 | 1.00E-200 | 0.85 |
|  |  | rs6117854 | G | A | 0.038 | 8.44E-05 | 2.60E-90 | 0.51 |
|  |  | rs614802 | A | G | -0.015 | -1.09E-04 | 1.30E-14 | 0.4 |
|  |  | rs61918361 | C | T | 0.011 | -1.87E-05 | 2.20E-09 | 0.88 |
|  |  | rs61921611 | T | C | -0.019 | 2.39E-05 | 9.40E-22 | 0.85 |
|  |  | rs62162671 | C | T | 0.013 | 2.06E-04 | 2.50E-12 | 0.1 |
|  |  | rs62228067 | A | C | -0.02 | -2.64E-05 | 1.00E-21 | 0.85 |
|  |  | rs62444275 | G | A | -0.064 | 4.40E-05 | 1.00E-200 | 0.72 |
|  |  | rs630510 | A | G | 0.011 | -4.63E-05 | 3.40E-10 | 0.7 |
|  |  | rs6427847 | A | G | 0.016 | -1.57E-04 | 7.30E-20 | 0.2 |
|  |  | rs6459983 | T | C | 0.01 | 9.84E-05 | 7.90E-09 | 0.41 |
|  |  | rs6461187 | C | A | 0.013 | 1.86E-05 | 1.30E-11 | 0.89 |
|  |  | rs6542920 | A | G | -0.012 | 2.88E-04 | 7.90E-11 | 0.021 |
|  |  | rs6546334 | C | T | 0.018 | -8.28E-05 | 1.90E-20 | 0.51 |
|  |  | rs666592 | C | T | 0.013 | -2.79E-04 | 1.00E-12 | 0.021 |
|  |  | rs6722557 | G | C | -0.02 | 3.10E-05 | 8.50E-20 | 0.82 |
|  |  | rs6751325 | G | T | 0.015 | 1.40E-04 | 2.00E-16 | 0.27 |
|  |  | rs6759927 | A | G | -0.019 | 3.62E-04 | 6.20E-26 | 0.005 |
|  |  | rs6782178 | C | T | -0.014 | -1.02E-04 | 4.50E-16 | 0.4 |
|  |  | rs6784925 | C | T | 0.011 | 1.19E-04 | 1.30E-09 | 0.32 |
|  |  | rs6786608 | G | A | -0.012 | -9.49E-05 | 1.20E-10 | 0.45 |
|  |  | rs6803861 | G | A | 0.017 | 1.77E-05 | 1.90E-18 | 0.89 |
|  |  | rs6864688 | C | T | 0.015 | 5.44E-05 | 2.60E-16 | 0.65 |
|  |  | rs6871819 | A | G | -0.012 | -1.56E-04 | 4.90E-11 | 0.2 |
|  |  | rs689411 | G | A | -0.015 | -5.51E-05 | 9.10E-16 | 0.66 |
|  |  | rs6938070 | T | A | 0.024 | 3.23E-04 | 3.20E-33 | 0.013 |
|  |  | rs6977460 | A | G | 0.017 | -1.86E-04 | 2.30E-16 | 0.17 |
|  |  | rs7000279 | C | T | -0.013 | 1.08E-04 | 2.10E-10 | 0.43 |
|  |  | rs7014448 | T | G | 0.025 | -1.92E-05 | 2.20E-39 | 0.88 |
|  |  | rs7017252 | C | T | -0.017 | 1.43E-04 | 9.20E-18 | 0.24 |
|  |  | rs7021585 | G | A | -0.019 | -8.03E-05 | 1.70E-19 | 0.56 |
|  |  | rs7040344 | C | T | 0.028 | -1.90E-04 | 1.10E-48 | 0.13 |
|  |  | rs7102 | T | C | 0.02 | 6.37E-06 | 3.60E-26 | 0.96 |
|  |  | rs7118404 | G | A | 0.035 | 1.42E-04 | 4.60E-79 | 0.26 |
|  |  | rs7125361 | C | G | -0.034 | -8.69E-05 | 8.20E-81 | 0.47 |
|  |  | rs7135535 | G | A | 0.013 | 4.72E-06 | 1.50E-13 | 0.97 |
|  |  | rs7147775 | T | G | 0.018 | -2.88E-04 | 1.20E-20 | 0.016 |
|  |  | rs7175531 | T | C | -0.032 | -1.18E-04 | 2.30E-66 | 0.34 |
|  |  | rs7183056 | C | T | 0.011 | 3.38E-05 | 5.90E-10 | 0.78 |
|  |  | rs7191269 | A | G | 0.03 | 3.67E-05 | 1.60E-61 | 0.76 |
|  |  | rs7197197 | G | A | -0.015 | 8.72E-05 | 4.80E-17 | 0.47 |
|  |  | rs719726 | C | T | -0.058 | -1.00E-04 | 1.00E-200 | 0.41 |
|  |  | rs7209460 | C | T | -0.052 | 1.40E-05 | 2.00E-154 | 0.91 |
|  |  | rs7236090 | T | C | -0.013 | -6.93E-05 | 2.00E-13 | 0.56 |
|  |  | rs724629 | G | T | -0.01 | -1.87E-04 | 3.70E-08 | 0.14 |
|  |  | rs7247412 | T | C | -0.012 | -1.21E-04 | 1.20E-09 | 0.37 |
|  |  | rs72810976 | G | A | -0.011 | -3.66E-06 | 2.20E-09 | 0.98 |
|  |  | rs72832241 | G | A | -0.019 | -1.61E-04 | 4.40E-18 | 0.25 |
|  |  | rs7290979 | A | C | 0.024 | 1.18E-05 | 6.70E-37 | 0.93 |
|  |  | rs7317781 | A | G | 0.015 | -7.48E-05 | 5.40E-14 | 0.56 |
|  |  | rs7368383 | T | C | 0.015 | -1.28E-04 | 1.70E-13 | 0.34 |
|  |  | rs737524 | A | C | 0.017 | 5.84E-05 | 5.80E-20 | 0.63 |
|  |  | rs7463837 | C | T | 0.012 | -1.31E-05 | 2.20E-08 | 0.92 |
|  |  | rs7484147 | T | C | 0.047 | -7.11E-05 | 6.40E-139 | 0.57 |
|  |  | rs7488974 | G | A | -0.051 | -4.30E-05 | 8.70E-175 | 0.72 |
|  |  | rs7504492 | T | C | -0.013 | 2.43E-04 | 6.50E-13 | 0.048 |
|  |  | rs7527300 | C | T | 0.025 | -2.76E-04 | 7.40E-43 | 0.023 |
|  |  | rs7577569 | G | A | -0.013 | 1.22E-04 | 2.20E-12 | 0.34 |
|  |  | rs7582828 | G | T | 0.021 | -8.85E-05 | 1.60E-24 | 0.52 |
|  |  | rs7585120 | C | T | -0.018 | 1.10E-04 | 1.10E-16 | 0.42 |
|  |  | rs7599234 | G | T | 0.012 | -3.18E-05 | 3.80E-12 | 0.79 |
|  |  | rs760402 | G | A | -0.012 | -2.40E-04 | 9.10E-10 | 0.063 |
|  |  | rs7646519 | G | A | -0.015 | -1.06E-05 | 1.40E-14 | 0.93 |
|  |  | rs7661259 | T | C | 0.014 | -5.69E-05 | 7.00E-13 | 0.64 |
|  |  | rs76846129 | T | A | -0.011 | 1.43E-04 | 1.90E-09 | 0.26 |
|  |  | rs7699606 | A | G | -0.012 | 7.01E-05 | 2.30E-09 | 0.61 |
|  |  | rs7703751 | A | T | 0.024 | 1.71E-04 | 1.70E-30 | 0.21 |
|  |  | rs77112003 | C | T | -0.014 | 7.94E-05 | 9.50E-13 | 0.54 |
|  |  | rs772175 | G | A | -0.01 | 6.62E-05 | 1.70E-08 | 0.6 |
|  |  | rs7728907 | G | A | 0.019 | -1.79E-04 | 1.70E-19 | 0.2 |
|  |  | rs7752642 | G | A | -0.018 | 9.24E-05 | 2.80E-22 | 0.44 |
|  |  | rs7771496 | C | T | 0.014 | -1.61E-04 | 4.10E-13 | 0.19 |
|  |  | rs7778978 | G | A | -0.012 | 8.44E-05 | 1.40E-09 | 0.52 |
|  |  | rs7789880 | C | T | 0.027 | -2.64E-04 | 2.70E-41 | 0.049 |
|  |  | rs7800633 | C | T | 0.011 | 1.24E-05 | 3.00E-11 | 0.92 |
|  |  | rs7814941 | A | G | -0.021 | 1.39E-04 | 1.30E-21 | 0.32 |
|  |  | rs7818431 | G | C | -0.01 | 5.09E-05 | 8.20E-09 | 0.67 |
|  |  | rs785836 | C | T | -0.012 | 1.24E-04 | 5.60E-11 | 0.32 |
|  |  | rs7866211 | T | C | 0.025 | -2.65E-05 | 6.00E-35 | 0.84 |
|  |  | rs7870885 | A | G | -0.015 | 2.01E-06 | 3.60E-14 | 0.99 |
|  |  | rs7898342 | G | A | -0.012 | 1.80E-05 | 3.20E-11 | 0.88 |
|  |  | rs7902719 | C | A | -0.011 | 6.44E-05 | 2.00E-09 | 0.6 |
|  |  | rs79730878 | T | C | 0.024 | 1.04E-04 | 2.20E-28 | 0.46 |
|  |  | rs8002850 | G | A | 0.029 | 3.90E-05 | 6.50E-52 | 0.76 |
|  |  | rs8008406 | T | C | 0.017 | -2.02E-04 | 5.80E-16 | 0.14 |
|  |  | rs8073697 | A | T | 0.014 | -1.55E-05 | 1.70E-14 | 0.9 |
|  |  | rs8114050 | A | G | 0.011 | 1.02E-04 | 1.30E-09 | 0.4 |
|  |  | rs8131741 | G | T | 0.019 | -7.53E-05 | 7.20E-26 | 0.53 |
|  |  | rs8180282 | T | C | -0.012 | 4.59E-05 | 4.50E-09 | 0.74 |
|  |  | rs8192803 | G | A | -0.011 | -9.73E-05 | 2.30E-08 | 0.42 |
|  |  | rs825453 | A | T | 0.016 | -2.06E-05 | 6.70E-20 | 0.87 |
|  |  | rs847147 | G | A | 0.017 | -2.93E-05 | 3.50E-18 | 0.83 |
|  |  | rs853163 | G | A | -0.013 | 7.22E-05 | 7.00E-11 | 0.6 |
|  |  | rs868127 | C | T | 0.017 | -1.58E-05 | 1.70E-15 | 0.91 |
|  |  | rs878522 | G | A | -0.011 | -5.85E-05 | 8.10E-09 | 0.65 |
|  |  | rs884205 | A | C | -0.026 | 6.23E-05 | 4.10E-36 | 0.65 |
|  |  | rs890074 | G | A | -0.012 | 1.23E-04 | 4.40E-12 | 0.3 |
|  |  | rs899631 | G | T | -0.018 | 1.74E-04 | 1.60E-21 | 0.15 |
|  |  | rs910405 | G | A | 0.013 | 4.84E-05 | 3.90E-11 | 0.72 |
|  |  | rs917441 | C | T | -0.029 | 1.01E-04 | 7.10E-57 | 0.42 |
|  |  | rs9257703 | T | G | 0.02 | 1.52E-04 | 6.00E-21 | 0.27 |
|  |  | rs930785 | T | A | -0.025 | -1.22E-04 | 2.80E-42 | 0.31 |
|  |  | rs9324890 | A | G | -0.012 | -5.90E-05 | 9.60E-09 | 0.66 |
|  |  | rs932828 | G | A | 0.032 | 9.82E-06 | 1.40E-63 | 0.94 |
|  |  | rs9447004 | A | G | 0.024 | -1.42E-04 | 2.10E-42 | 0.23 |
|  |  | rs9466072 | C | G | -0.01 | -3.44E-04 | 6.20E-09 | 0.005 |
|  |  | rs947091 | G | A | -0.04 | 1.01E-04 | 8.40E-112 | 0.4 |
|  |  | rs9513510 | G | C | 0.035 | -1.90E-04 | 6.60E-73 | 0.14 |
|  |  | rs9521510 | T | C | 0.021 | -2.02E-04 | 2.50E-29 | 0.11 |
|  |  | rs9523981 | C | T | 0.015 | -4.66E-04 | 2.80E-17 | 0 |
|  |  | rs9530279 | T | C | 0.019 | 1.36E-04 | 5.20E-20 | 0.31 |
|  |  | rs9545557 | G | A | -0.01 | -2.16E-04 | 3.20E-09 | 0.07 |
|  |  | rs957772 | A | G | 0.012 | -5.92E-05 | 2.00E-11 | 0.62 |
|  |  | rs9594738 | C | T | 0.048 | -7.05E-06 | 1.00E-152 | 0.95 |
|  |  | rs960192 | C | T | -0.017 | 7.12E-05 | 2.80E-21 | 0.56 |
|  |  | rs9612225 | G | A | 0.011 | 2.35E-04 | 3.90E-08 | 0.082 |
|  |  | rs9725597 | G | A | -0.015 | 1.44E-05 | 4.50E-13 | 0.92 |
|  |  | rs9811191 | T | C | -0.014 | 5.95E-05 | 9.10E-15 | 0.62 |
|  |  | rs984409 | G | A | 0.012 | -1.16E-04 | 1.50E-10 | 0.35 |
|  |  | rs9896306 | C | A | 0.026 | -1.12E-04 | 1.00E-38 | 0.4 |
|  |  | rs9907483 | A | G | 0.017 | 4.41E-05 | 1.60E-18 | 0.73 |
|  |  | rs9921222 | C | T | 0.026 | 2.05E-04 | 7.60E-45 | 0.085 |
| FA-BMD | RA | rs13423976 | A | G | -0.098 | -5.65E-05 | 2.30E-08 | 0.67 |
|  |  | rs6894139 | G | T | -0.089 | 2.61E-04 | 2.52E-08 | 0.029 |
|  |  | rs7776725 | C | T | 0.186 | -3.72E-05 | 1.21E-25 | 0.78 |
| FN-BMD | RA | rs10170839 | C | A | -0.059 | 5.34E-05 | 1.20E-14 | 0.65 |
|  |  | rs10794639 | G | A | -0.051 | -2.06E-04 | 3.30E-11 | 0.084 |
|  |  | rs10946458 | C | T | -0.045 | -4.52E-04 | 3.63E-08 | 0 |
|  |  | rs13194508 | C | T | -0.052 | 9.69E-06 | 1.30E-08 | 0.94 |
|  |  | rs1366594 | C | A | -0.079 | 2.67E-04 | 5.44E-25 | 0.025 |
|  |  | rs1485307 | T | C | 0.062 | 1.29E-05 | 2.49E-15 | 0.91 |
|  |  | rs1785493 | T | C | -0.045 | 1.96E-04 | 4.06E-08 | 0.12 |
|  |  | rs2566752 | C | T | 0.062 | 3.55E-05 | 3.65E-15 | 0.77 |
|  |  | rs3779381 | G | A | 0.058 | -1.18E-04 | 2.87E-11 | 0.38 |
|  |  | rs436448 | T | C | -0.064 | -2.38E-05 | 1.56E-16 | 0.84 |
|  |  | rs4448201 | G | C | -0.066 | 1.08E-04 | 4.37E-16 | 0.39 |
|  |  | rs4759320 | C | G | -0.045 | -1.96E-05 | 3.33E-08 | 0.88 |
|  |  | rs7209460 | C | T | -0.051 | 1.40E-05 | 1.35E-09 | 0.91 |
|  |  | rs9478217 | A | G | -0.053 | 2.63E-05 | 1.23E-11 | 0.83 |
| LS-BMD | RA | rs1023940 | A | G | 0.065 | 7.00E-05 | 6.47E-13 | 0.56 |
|  |  | rs11680288 | G | A | 0.054 | -1.18E-04 | 3.12E-09 | 0.32 |
|  |  | rs13046645 | A | T | -0.056 | 8.27E-05 | 2.92E-08 | 0.53 |
|  |  | rs1357651 | T | G | -0.068 | -1.43E-04 | 3.75E-13 | 0.25 |
|  |  | rs2220189 | C | G | 0.083 | 3.46E-05 | 4.25E-20 | 0.77 |
|  |  | rs2235811 | G | A | -0.054 | -3.29E-04 | 4.66E-09 | 0.006 |
|  |  | rs2291467 | T | C | -0.077 | 2.89E-04 | 9.64E-14 | 0.033 |
|  |  | rs2566752 | C | T | 0.083 | 3.55E-05 | 1.49E-19 | 0.77 |
|  |  | rs35681117 | T | C | 0.055 | -1.81E-04 | 2.39E-08 | 0.17 |
|  |  | rs401680 | A | T | -0.057 | 3.43E-05 | 3.70E-10 | 0.77 |
|  |  | rs6965122 | G | A | -0.062 | 7.50E-05 | 7.40E-11 | 0.55 |
|  |  | rs7807953 | T | C | 0.075 | -9.49E-05 | 4.11E-14 | 0.48 |
|  |  | rs884205 | A | C | -0.062 | 6.23E-05 | 2.77E-09 | 0.65 |
|  |  | rs894738 | A | G | -0.063 | 2.63E-05 | 2.00E-11 | 0.83 |
|  |  | rs9533094 | G | A | -0.083 | 1.32E-05 | 2.80E-20 | 0.91 |
|  |  | rs9921222 | T | C | -0.053 | -2.05E-04 | 3.16E-09 | 0.085 |
| TB-BMD | RA | rs10048745 | A | G | -0.039 | 4.41E-05 | 6.44E-09 | 0.75 |
|  |  | rs1037011 | T | C | -0.04 | -1.36E-04 | 1.54E-12 | 0.25 |
|  |  | rs10735851 | A | G | -0.054 | -1.55E-05 | 5.84E-18 | 0.91 |
|  |  | rs10777212 | T | G | 0.045 | 4.27E-05 | 5.05E-14 | 0.73 |
|  |  | rs10788264 | A | G | -0.034 | 2.78E-04 | 2.61E-09 | 0.021 |
|  |  | rs10875906 | T | C | 0.051 | -1.40E-04 | 1.85E-13 | 0.3 |
|  |  | rs10901216 | A | G | -0.047 | 1.73E-04 | 5.53E-15 | 0.17 |
|  |  | rs11228240 | T | C | -0.083 | 3.17E-04 | 1.72E-35 | 0.018 |
|  |  | rs11745493 | A | G | 0.045 | 1.67E-04 | 7.75E-12 | 0.22 |
|  |  | rs11898505 | A | G | 0.034 | -9.20E-05 | 1.28E-08 | 0.46 |
|  |  | rs11904127 | A | G | -0.032 | -7.09E-05 | 1.18E-08 | 0.56 |
|  |  | rs11934731 | A | G | -0.067 | 1.06E-04 | 8.39E-29 | 0.41 |
|  |  | rs11995824 | C | G | 0.068 | 2.30E-05 | 1.06E-31 | 0.85 |
|  |  | rs12534510 | A | C | -0.04 | 6.20E-05 | 3.15E-12 | 0.6 |
|  |  | rs13204965 | A | C | 0.062 | -3.10E-05 | 1.02E-18 | 0.82 |
|  |  | rs1452102 | T | G | -0.035 | -1.37E-04 | 1.74E-09 | 0.26 |
|  |  | rs1548607 | A | G | 0.036 | -5.19E-05 | 4.18E-08 | 0.68 |
|  |  | rs2252865 | T | C | -0.033 | 9.71E-05 | 4.72E-08 | 0.43 |
|  |  | rs2553773 | C | G | -0.037 | 1.21E-04 | 1.49E-10 | 0.32 |
|  |  | rs2566752 | T | C | -0.072 | -3.55E-05 | 1.88E-34 | 0.77 |
|  |  | rs2873195 | A | T | -0.041 | 2.10E-05 | 4.31E-11 | 0.87 |
|  |  | rs34102936 | A | G | 0.047 | 5.27E-05 | 1.87E-16 | 0.66 |
|  |  | rs35125553 | A | G | -0.038 | -9.76E-05 | 5.20E-09 | 0.46 |
|  |  | rs35199438 | T | G | -0.049 | 1.34E-05 | 2.36E-15 | 0.92 |
|  |  | rs3743347 | A | C | 0.052 | 1.28E-04 | 1.75E-14 | 0.36 |
|  |  | rs3801387 | A | G | -0.135 | 8.84E-05 | 1.15E-100 | 0.51 |
|  |  | rs447911 | C | G | 0.071 | -3.44E-05 | 6.29E-36 | 0.77 |
|  |  | rs55781332 | A | G | -0.055 | -7.25E-06 | 8.07E-16 | 0.96 |
|  |  | rs6029130 | T | C | 0.035 | 1.43E-04 | 3.50E-08 | 0.29 |
|  |  | rs6040063 | A | G | 0.036 | 3.21E-04 | 1.78E-10 | 0.007 |
|  |  | rs61837366 | T | C | 0.042 | 4.51E-05 | 3.07E-09 | 0.73 |
|  |  | rs633995 | A | G | 0.035 | 2.58E-04 | 1.61E-09 | 0.035 |
|  |  | rs634277 | A | G | 0.061 | 2.84E-05 | 2.15E-23 | 0.82 |
|  |  | rs6465511 | C | G | -0.074 | 8.39E-05 | 1.03E-34 | 0.5 |
|  |  | rs6557155 | T | G | -0.075 | 5.45E-05 | 2.56E-37 | 0.66 |
|  |  | rs6960249 | T | G | 0.033 | -1.23E-05 | 1.45E-08 | 0.92 |
|  |  | rs7105860 | C | G | -0.047 | 6.38E-05 | 2.36E-15 | 0.61 |
|  |  | rs725670 | A | G | -0.032 | -2.31E-05 | 3.61E-08 | 0.85 |
|  |  | rs73305797 | A | T | 0.042 | -5.93E-05 | 2.40E-10 | 0.66 |
|  |  | rs7548588 | T | C | -0.037 | -1.59E-04 | 2.21E-10 | 0.19 |
|  |  | rs757138 | T | G | -0.035 | 5.05E-05 | 3.33E-08 | 0.71 |
|  |  | rs7586085 | A | G | 0.053 | -6.13E-05 | 8.64E-21 | 0.61 |
|  |  | rs7728694 | T | G | -0.05 | 2.20E-04 | 1.30E-17 | 0.065 |
|  |  | rs7741085 | T | C | 0.042 | -1.31E-04 | 1.51E-13 | 0.28 |
|  |  | rs780096 | C | G | -0.031 | 1.21E-04 | 4.58E-08 | 0.31 |
|  |  | rs8047501 | A | G | 0.052 | 2.13E-04 | 1.13E-18 | 0.077 |
|  |  | rs8070128 | T | C | -0.039 | -2.06E-05 | 1.98E-11 | 0.87 |
|  |  | rs818427 | T | C | 0.034 | 2.61E-05 | 2.37E-08 | 0.84 |
|  |  | rs838721 | A | G | -0.031 | -1.39E-04 | 4.48E-08 | 0.25 |
|  |  | rs884205 | A | C | -0.053 | 6.23E-05 | 4.39E-15 | 0.65 |
|  |  | rs9594738 | T | C | -0.061 | 7.05E-06 | 3.84E-27 | 0.95 |
|  |  | rs9910055 | T | C | 0.044 | -3.26E-04 | 3.12E-11 | 0.021 |
|  |  | rs9972944 | A | G | 0.036 | 5.83E-05 | 6.87E-10 | 0.64 |
|  |  | rs9976876 | T | G | -0.038 | 7.85E-05 | 8.01E-11 | 0.51 |

# IVs represents instruments variates; MR represents Mendelian randomization；FA-, FN-, LS-, TB-BMD represent forearm, femoral neck, lumbar spine and total body BMD respectively; RA represents rheumatic arthritis.
