# Supplementary material for: Rheumatoid arthritis and osteoporosis: a bi-directional Mendelian randomization study
Source: Aging (Albany NY). 2021 May 18;13(10):14109–30. doi: 10.18632/aging.203029 (PMC8202858; doi:10.18632/aging.203029)
Supplement: Supplementary Table 2 [file aging-13-203029-s003.docx]

**Supplementary Table 2. IVs for MR analyses in negative control.**

| **SNP** | **exposure** | **outcome** | **beta.exposure** | **se.exposure** | **beta.outcome** | **se.outcome** |
| --- | --- | --- | --- | --- | --- | --- |
| rs10022648 | Heel BMD | Myopia | 0.016 | 0.002 | 0.011 | 0.060 |
| rs10063744 | Heel BMD | Myopia | 0.013 | 0.002 | -0.024 | 0.059 |
| rs10197427 | Heel BMD | Myopia | 0.057 | 0.002 | -0.113 | 0.068 |
| rs10279360 | Heel BMD | Myopia | 0.010 | 0.002 | -0.003 | 0.066 |
| rs1029830 | Heel BMD | Myopia | 0.013 | 0.002 | -0.023 | 0.060 |
| rs10407062 | Heel BMD | Myopia | 0.014 | 0.002 | 0.018 | 0.058 |
| rs1043003 | Heel BMD | Myopia | 0.017 | 0.002 | -0.050 | 0.065 |
| rs10515269 | Heel BMD | Myopia | -0.015 | 0.002 | -0.029 | 0.058 |
| rs1056322 | Heel BMD | Myopia | 0.013 | 0.002 | 0.020 | 0.061 |
| rs10750000 | Heel BMD | Myopia | 0.056 | 0.002 | -0.016 | 0.063 |
| rs10763557 | Heel BMD | Myopia | 0.019 | 0.002 | 0.071 | 0.058 |
| rs10764201 | Heel BMD | Myopia | -0.020 | 0.002 | 0.054 | 0.091 |
| rs10765568 | Heel BMD | Myopia | 0.017 | 0.002 | -0.076 | 0.060 |
| rs10779795 | Heel BMD | Myopia | 0.020 | 0.002 | 0.117 | 0.064 |
| rs10780789 | Heel BMD | Myopia | -0.012 | 0.002 | -0.058 | 0.058 |
| rs10788221 | Heel BMD | Myopia | -0.017 | 0.002 | -0.167 | 0.063 |
| rs10842704 | Heel BMD | Myopia | -0.028 | 0.002 | 0.121 | 0.064 |
| rs10859561 | Heel BMD | Myopia | -0.016 | 0.002 | 0.016 | 0.058 |
| rs10882165 | Heel BMD | Myopia | -0.013 | 0.002 | -0.028 | 0.060 |
| rs10885434 | Heel BMD | Myopia | 0.022 | 0.002 | 0.013 | 0.065 |
| rs10920352 | Heel BMD | Myopia | -0.011 | 0.002 | -0.035 | 0.058 |
| rs10930953 | Heel BMD | Myopia | 0.019 | 0.002 | 0.009 | 0.060 |
| rs11048374 | Heel BMD | Myopia | -0.012 | 0.002 | 0.005 | 0.058 |
| rs11050203 | Heel BMD | Myopia | -0.013 | 0.002 | -0.072 | 0.068 |
| rs11054754 | Heel BMD | Myopia | 0.014 | 0.002 | 0.027 | 0.058 |
| rs11083780 | Heel BMD | Myopia | 0.011 | 0.002 | -0.038 | 0.060 |
| rs11170800 | Heel BMD | Myopia | -0.011 | 0.002 | -0.020 | 0.063 |
| rs11175835 | Heel BMD | Myopia | 0.023 | 0.002 | 0.060 | 0.069 |
| rs11191149 | Heel BMD | Myopia | 0.012 | 0.002 | -0.019 | 0.063 |
| rs11228240 | Heel BMD | Myopia | 0.043 | 0.002 | -0.123 | 0.076 |
| rs11238754 | Heel BMD | Myopia | -0.012 | 0.002 | -0.049 | 0.058 |
| rs11242774 | Heel BMD | Myopia | 0.014 | 0.002 | -0.035 | 0.059 |
| rs11632673 | Heel BMD | Myopia | -0.023 | 0.002 | -0.075 | 0.058 |
| rs11643240 | Heel BMD | Myopia | 0.015 | 0.002 | -0.073 | 0.066 |
| rs11672782 | Heel BMD | Myopia | -0.010 | 0.002 | 0.067 | 0.058 |
| rs11688492 | Heel BMD | Myopia | 0.013 | 0.002 | -0.009 | 0.060 |
| rs11719201 | Heel BMD | Myopia | 0.013 | 0.002 | 0.022 | 0.076 |
| rs11737408 | Heel BMD | Myopia | 0.011 | 0.002 | -0.032 | 0.068 |
| rs11880992 | Heel BMD | Myopia | 0.017 | 0.002 | -0.003 | 0.060 |
| rs11894900 | Heel BMD | Myopia | -0.015 | 0.002 | 0.028 | 0.061 |
| rs11934731 | Heel BMD | Myopia | 0.036 | 0.002 | -0.027 | 0.064 |
| rs11935978 | Heel BMD | Myopia | 0.011 | 0.002 | -0.076 | 0.059 |
| rs12077443 | Heel BMD | Myopia | -0.010 | 0.002 | 0.033 | 0.058 |
| rs12121554 | Heel BMD | Myopia | -0.024 | 0.002 | 0.030 | 0.061 |
| rs12149673 | Heel BMD | Myopia | -0.029 | 0.002 | 0.051 | 0.065 |
| rs12210993 | Heel BMD | Myopia | 0.022 | 0.002 | -0.028 | 0.057 |
| rs12276167 | Heel BMD | Myopia | 0.014 | 0.002 | -0.016 | 0.061 |
| rs12346689 | Heel BMD | Myopia | 0.019 | 0.002 | 0.093 | 0.061 |
| rs12372718 | Heel BMD | Myopia | 0.015 | 0.002 | -0.075 | 0.061 |
| rs12414040 | Heel BMD | Myopia | -0.011 | 0.002 | 0.005 | 0.058 |
| rs12443188 | Heel BMD | Myopia | -0.022 | 0.002 | -0.011 | 0.064 |
| rs1255471 | Heel BMD | Myopia | -0.010 | 0.002 | -0.014 | 0.063 |
| rs1256065 | Heel BMD | Myopia | -0.012 | 0.002 | -0.071 | 0.058 |
| rs12587727 | Heel BMD | Myopia | -0.011 | 0.002 | -0.050 | 0.074 |
| rs12614693 | Heel BMD | Myopia | 0.014 | 0.002 | 0.097 | 0.080 |
| rs12683791 | Heel BMD | Myopia | -0.029 | 0.002 | -0.035 | 0.061 |
| rs12703135 | Heel BMD | Myopia | 0.012 | 0.002 | 0.123 | 0.066 |
| rs12733821 | Heel BMD | Myopia | -0.016 | 0.002 | -0.091 | 0.060 |
| rs12811685 | Heel BMD | Myopia | 0.018 | 0.002 | -0.046 | 0.064 |
| rs12932885 | Heel BMD | Myopia | 0.024 | 0.002 | -0.025 | 0.058 |
| rs12973365 | Heel BMD | Myopia | -0.010 | 0.002 | -0.059 | 0.058 |
| rs12974515 | Heel BMD | Myopia | 0.010 | 0.002 | 0.054 | 0.058 |
| rs12986939 | Heel BMD | Myopia | 0.050 | 0.002 | 0.080 | 0.068 |
| rs13002567 | Heel BMD | Myopia | -0.018 | 0.002 | 0.089 | 0.062 |
| rs13022378 | Heel BMD | Myopia | -0.023 | 0.002 | -0.067 | 0.059 |
| rs13065094 | Heel BMD | Myopia | -0.021 | 0.002 | -0.054 | 0.058 |
| rs13070996 | Heel BMD | Myopia | 0.014 | 0.002 | -0.036 | 0.062 |
| rs13083728 | Heel BMD | Myopia | -0.017 | 0.002 | 0.051 | 0.066 |
| rs13088318 | Heel BMD | Myopia | -0.015 | 0.002 | 0.026 | 0.059 |
| rs13154707 | Heel BMD | Myopia | -0.021 | 0.002 | -0.010 | 0.063 |
| rs13230922 | Heel BMD | Myopia | 0.015 | 0.002 | -0.003 | 0.061 |
| rs13427681 | Heel BMD | Myopia | 0.011 | 0.002 | -0.015 | 0.061 |
| rs134622 | Heel BMD | Myopia | 0.040 | 0.002 | -0.002 | 0.067 |
| rs1392788 | Heel BMD | Myopia | 0.011 | 0.002 | -0.037 | 0.058 |
| rs1426889 | Heel BMD | Myopia | -0.012 | 0.002 | 0.038 | 0.058 |
| rs1455420 | Heel BMD | Myopia | -0.010 | 0.002 | -0.140 | 0.058 |
| rs1455816 | Heel BMD | Myopia | 0.012 | 0.002 | -0.076 | 0.064 |
| rs1463597 | Heel BMD | Myopia | -0.012 | 0.002 | -0.008 | 0.058 |
| rs1468316 | Heel BMD | Myopia | -0.010 | 0.002 | -0.140 | 0.065 |
| rs1484434 | Heel BMD | Myopia | 0.012 | 0.002 | -0.058 | 0.063 |
| rs1500920 | Heel BMD | Myopia | 0.016 | 0.002 | -0.007 | 0.060 |
| rs1502201 | Heel BMD | Myopia | 0.017 | 0.002 | 0.028 | 0.060 |
| rs1533142 | Heel BMD | Myopia | 0.023 | 0.002 | 0.132 | 0.063 |
| rs1535571 | Heel BMD | Myopia | -0.013 | 0.002 | 0.103 | 0.059 |
| rs1548607 | Heel BMD | Myopia | 0.018 | 0.002 | 0.065 | 0.061 |
| rs1550270 | Heel BMD | Myopia | -0.016 | 0.002 | 0.161 | 0.064 |
| rs1564983 | Heel BMD | Myopia | -0.022 | 0.002 | 0.045 | 0.061 |
| rs1622638 | Heel BMD | Myopia | -0.015 | 0.002 | 0.160 | 0.058 |
| rs167365 | Heel BMD | Myopia | 0.019 | 0.002 | 0.023 | 0.064 |
| rs16884419 | Heel BMD | Myopia | 0.014 | 0.002 | -0.139 | 0.067 |
| rs1706708 | Heel BMD | Myopia | 0.029 | 0.002 | 0.075 | 0.064 |
| rs174598 | Heel BMD | Myopia | -0.014 | 0.002 | -0.031 | 0.061 |
| rs17684825 | Heel BMD | Myopia | -0.012 | 0.002 | -0.063 | 0.059 |
| rs17700633 | Heel BMD | Myopia | -0.013 | 0.002 | -0.021 | 0.065 |
| rs1777277 | Heel BMD | Myopia | 0.011 | 0.002 | 0.067 | 0.058 |
| rs17796692 | Heel BMD | Myopia | 0.018 | 0.002 | 0.111 | 0.060 |
| rs178896 | Heel BMD | Myopia | 0.016 | 0.002 | 0.046 | 0.065 |
| rs1844776 | Heel BMD | Myopia | -0.012 | 0.002 | 0.050 | 0.058 |
| rs1861000 | Heel BMD | Myopia | -0.062 | 0.002 | 0.026 | 0.061 |
| rs1865712 | Heel BMD | Myopia | -0.012 | 0.002 | 0.025 | 0.067 |
| rs1897465 | Heel BMD | Myopia | 0.013 | 0.002 | 0.007 | 0.063 |
| rs1999536 | Heel BMD | Myopia | -0.021 | 0.002 | -0.031 | 0.058 |
| rs2005617 | Heel BMD | Myopia | -0.013 | 0.002 | 0.131 | 0.058 |
| rs2052480 | Heel BMD | Myopia | -0.027 | 0.002 | 0.040 | 0.072 |
| rs2069442 | Heel BMD | Myopia | 0.019 | 0.002 | 0.110 | 0.065 |
| rs2085490 | Heel BMD | Myopia | -0.013 | 0.002 | -0.053 | 0.058 |
| rs210374 | Heel BMD | Myopia | -0.014 | 0.002 | -0.052 | 0.059 |
| rs2174633 | Heel BMD | Myopia | -0.016 | 0.002 | 0.076 | 0.061 |
| rs2179519 | Heel BMD | Myopia | 0.011 | 0.002 | -0.020 | 0.064 |
| rs2239626 | Heel BMD | Myopia | -0.017 | 0.002 | -0.033 | 0.069 |
| rs2240226 | Heel BMD | Myopia | -0.034 | 0.002 | -0.068 | 0.063 |
| rs2254027 | Heel BMD | Myopia | -0.019 | 0.002 | 0.012 | 0.060 |
| rs2302407 | Heel BMD | Myopia | 0.016 | 0.002 | -0.027 | 0.058 |
| rs2337106 | Heel BMD | Myopia | -0.019 | 0.002 | 0.059 | 0.058 |
| rs2353551 | Heel BMD | Myopia | -0.011 | 0.002 | -0.006 | 0.068 |
| rs2374653 | Heel BMD | Myopia | 0.012 | 0.002 | 0.060 | 0.059 |
| rs2382801 | Heel BMD | Myopia | -0.020 | 0.002 | -0.058 | 0.058 |
| rs2388792 | Heel BMD | Myopia | -0.013 | 0.002 | 0.008 | 0.059 |
| rs241770 | Heel BMD | Myopia | -0.012 | 0.002 | 0.116 | 0.060 |
| rs2430689 | Heel BMD | Myopia | 0.016 | 0.002 | -0.070 | 0.062 |
| rs2546985 | Heel BMD | Myopia | 0.015 | 0.002 | -0.028 | 0.071 |
| rs2553772 | Heel BMD | Myopia | -0.028 | 0.002 | -0.035 | 0.058 |
| rs258223 | Heel BMD | Myopia | 0.011 | 0.002 | 0.030 | 0.061 |
| rs2639953 | Heel BMD | Myopia | 0.028 | 0.002 | 0.099 | 0.058 |
| rs264647 | Heel BMD | Myopia | -0.020 | 0.002 | 0.013 | 0.058 |
| rs2722176 | Heel BMD | Myopia | -0.013 | 0.002 | -0.010 | 0.063 |
| rs2761884 | Heel BMD | Myopia | 0.050 | 0.002 | 0.057 | 0.058 |
| rs277402 | Heel BMD | Myopia | -0.013 | 0.002 | -0.041 | 0.063 |
| rs2830913 | Heel BMD | Myopia | -0.024 | 0.002 | 0.031 | 0.061 |
| rs28364580 | Heel BMD | Myopia | 0.022 | 0.002 | 0.074 | 0.068 |
| rs28557305 | Heel BMD | Myopia | 0.026 | 0.002 | -0.036 | 0.060 |
| rs2929308 | Heel BMD | Myopia | 0.044 | 0.002 | -0.026 | 0.059 |
| rs2982571 | Heel BMD | Myopia | -0.077 | 0.002 | -0.014 | 0.058 |
| rs3131593 | Heel BMD | Myopia | 0.014 | 0.002 | -0.012 | 0.064 |
| rs314261 | Heel BMD | Myopia | -0.013 | 0.002 | -0.084 | 0.059 |
| rs330091 | Heel BMD | Myopia | -0.019 | 0.002 | 0.098 | 0.065 |
| rs34068557 | Heel BMD | Myopia | 0.012 | 0.002 | -0.027 | 0.061 |
| rs344035 | Heel BMD | Myopia | 0.015 | 0.002 | -0.040 | 0.058 |
| rs34435227 | Heel BMD | Myopia | 0.013 | 0.002 | 0.105 | 0.058 |
| rs34441013 | Heel BMD | Myopia | -0.032 | 0.002 | 0.177 | 0.067 |
| rs34627117 | Heel BMD | Myopia | -0.022 | 0.002 | -0.021 | 0.065 |
| rs34853396 | Heel BMD | Myopia | 0.012 | 0.002 | -0.014 | 0.059 |
| rs34879158 | Heel BMD | Myopia | -0.017 | 0.002 | 0.098 | 0.068 |
| rs35155027 | Heel BMD | Myopia | 0.016 | 0.002 | 0.082 | 0.063 |
| rs35657711 | Heel BMD | Myopia | -0.051 | 0.002 | -0.140 | 0.067 |
| rs35713954 | Heel BMD | Myopia | 0.011 | 0.002 | 0.014 | 0.059 |
| rs359942 | Heel BMD | Myopia | -0.010 | 0.002 | -0.002 | 0.058 |
| rs36016056 | Heel BMD | Myopia | -0.019 | 0.002 | 0.036 | 0.065 |
| rs36124395 | Heel BMD | Myopia | 0.010 | 0.002 | 0.012 | 0.063 |
| rs368510 | Heel BMD | Myopia | -0.024 | 0.002 | 0.156 | 0.060 |
| rs370387 | Heel BMD | Myopia | -0.047 | 0.002 | -0.099 | 0.058 |
| rs3747010 | Heel BMD | Myopia | -0.012 | 0.002 | 0.052 | 0.066 |
| rs3752075 | Heel BMD | Myopia | -0.010 | 0.002 | 0.078 | 0.059 |
| rs3760456 | Heel BMD | Myopia | 0.023 | 0.002 | -0.024 | 0.058 |
| rs3765971 | Heel BMD | Myopia | -0.028 | 0.002 | 0.003 | 0.060 |
| rs3777787 | Heel BMD | Myopia | 0.052 | 0.002 | 0.014 | 0.059 |
| rs3796014 | Heel BMD | Myopia | -0.051 | 0.002 | -0.062 | 0.073 |
| rs3828559 | Heel BMD | Myopia | 0.015 | 0.002 | 0.027 | 0.068 |
| rs3936185 | Heel BMD | Myopia | 0.020 | 0.002 | -0.045 | 0.060 |
| rs3941888 | Heel BMD | Myopia | -0.013 | 0.002 | -0.069 | 0.062 |
| rs4358110 | Heel BMD | Myopia | -0.014 | 0.002 | 0.064 | 0.072 |
| rs4360494 | Heel BMD | Myopia | -0.019 | 0.002 | 0.026 | 0.059 |
| rs4383904 | Heel BMD | Myopia | -0.045 | 0.002 | 0.097 | 0.063 |
| rs4401555 | Heel BMD | Myopia | 0.011 | 0.002 | 0.069 | 0.058 |
| rs4488059 | Heel BMD | Myopia | 0.017 | 0.002 | -0.012 | 0.060 |
| rs4595506 | Heel BMD | Myopia | 0.023 | 0.002 | -0.052 | 0.058 |
| rs4600907 | Heel BMD | Myopia | -0.015 | 0.002 | -0.003 | 0.065 |
| rs4635400 | Heel BMD | Myopia | 0.050 | 0.002 | -0.007 | 0.059 |
| rs4693374 | Heel BMD | Myopia | -0.019 | 0.002 | 0.027 | 0.059 |
| rs4694691 | Heel BMD | Myopia | -0.009 | 0.002 | 0.033 | 0.058 |
| rs4724126 | Heel BMD | Myopia | -0.017 | 0.002 | -0.011 | 0.062 |
| rs4739697 | Heel BMD | Myopia | 0.019 | 0.002 | -0.016 | 0.062 |
| rs4806862 | Heel BMD | Myopia | -0.020 | 0.002 | 0.040 | 0.059 |
| rs4810131 | Heel BMD | Myopia | 0.010 | 0.002 | 0.060 | 0.058 |
| rs4836373 | Heel BMD | Myopia | 0.019 | 0.002 | -0.041 | 0.059 |
| rs4876361 | Heel BMD | Myopia | 0.013 | 0.002 | 0.087 | 0.063 |
| rs4884522 | Heel BMD | Myopia | 0.011 | 0.002 | 0.033 | 0.059 |
| rs4886486 | Heel BMD | Myopia | -0.012 | 0.002 | 0.040 | 0.064 |
| rs4889490 | Heel BMD | Myopia | -0.012 | 0.002 | 0.033 | 0.059 |
| rs4960293 | Heel BMD | Myopia | 0.014 | 0.002 | 0.004 | 0.060 |
| rs55704141 | Heel BMD | Myopia | -0.029 | 0.002 | -0.070 | 0.059 |
| rs55709850 | Heel BMD | Myopia | 0.017 | 0.002 | 0.093 | 0.063 |
| rs55771168 | Heel BMD | Myopia | 0.011 | 0.002 | -0.025 | 0.063 |
| rs56371096 | Heel BMD | Myopia | -0.009 | 0.002 | -0.058 | 0.060 |
| rs571356 | Heel BMD | Myopia | 0.016 | 0.002 | 0.077 | 0.064 |
| rs5762768 | Heel BMD | Myopia | 0.020 | 0.002 | -0.010 | 0.058 |
| rs594647 | Heel BMD | Myopia | -0.010 | 0.002 | -0.125 | 0.058 |
| rs597319 | Heel BMD | Myopia | 0.066 | 0.002 | -0.076 | 0.063 |
| rs6117854 | Heel BMD | Myopia | 0.038 | 0.002 | -0.014 | 0.060 |
| rs614802 | Heel BMD | Myopia | -0.015 | 0.002 | -0.053 | 0.061 |
| rs61918361 | Heel BMD | Myopia | 0.011 | 0.002 | 0.037 | 0.058 |
| rs62162671 | Heel BMD | Myopia | 0.013 | 0.002 | 0.008 | 0.065 |
| rs62228067 | Heel BMD | Myopia | -0.020 | 0.002 | -0.141 | 0.075 |
| rs62444275 | Heel BMD | Myopia | -0.064 | 0.002 | -0.142 | 0.059 |
| rs630510 | Heel BMD | Myopia | 0.011 | 0.002 | -0.011 | 0.060 |
| rs6427847 | Heel BMD | Myopia | 0.016 | 0.002 | 0.025 | 0.058 |
| rs6542920 | Heel BMD | Myopia | -0.012 | 0.002 | -0.078 | 0.064 |
| rs6546334 | Heel BMD | Myopia | 0.018 | 0.002 | -0.060 | 0.058 |
| rs666592 | Heel BMD | Myopia | 0.013 | 0.002 | -0.027 | 0.061 |
| rs6722557 | Heel BMD | Myopia | -0.020 | 0.002 | 0.022 | 0.063 |
| rs6751325 | Heel BMD | Myopia | 0.015 | 0.002 | -0.005 | 0.065 |
| rs6759927 | Heel BMD | Myopia | -0.019 | 0.002 | 0.073 | 0.060 |
| rs6782178 | Heel BMD | Myopia | -0.014 | 0.002 | -0.014 | 0.060 |
| rs6784925 | Heel BMD | Myopia | 0.011 | 0.002 | 0.096 | 0.059 |
| rs6786608 | Heel BMD | Myopia | -0.012 | 0.002 | -0.011 | 0.061 |
| rs6864688 | Heel BMD | Myopia | 0.015 | 0.002 | 0.009 | 0.058 |
| rs689411 | Heel BMD | Myopia | -0.015 | 0.002 | -0.009 | 0.059 |
| rs6938070 | Heel BMD | Myopia | 0.024 | 0.002 | -0.048 | 0.068 |
| rs6977460 | Heel BMD | Myopia | 0.017 | 0.002 | 0.005 | 0.072 |
| rs7000279 | Heel BMD | Myopia | -0.013 | 0.002 | -0.062 | 0.066 |
| rs7014448 | Heel BMD | Myopia | 0.025 | 0.002 | -0.002 | 0.066 |
| rs7017252 | Heel BMD | Myopia | -0.017 | 0.002 | 0.026 | 0.062 |
| rs7021585 | Heel BMD | Myopia | -0.019 | 0.002 | 0.048 | 0.065 |
| rs7040344 | Heel BMD | Myopia | 0.028 | 0.002 | -0.040 | 0.058 |
| rs7118404 | Heel BMD | Myopia | 0.035 | 0.002 | 0.024 | 0.062 |
| rs7125361 | Heel BMD | Myopia | -0.034 | 0.002 | 0.039 | 0.058 |
| rs7175531 | Heel BMD | Myopia | -0.032 | 0.002 | -0.125 | 0.061 |
| rs7191269 | Heel BMD | Myopia | 0.030 | 0.002 | 0.064 | 0.060 |
| rs7197197 | Heel BMD | Myopia | -0.015 | 0.002 | -0.041 | 0.059 |
| rs7209460 | Heel BMD | Myopia | -0.052 | 0.002 | 0.033 | 0.062 |
| rs7236090 | Heel BMD | Myopia | -0.013 | 0.002 | 0.018 | 0.059 |
| rs724629 | Heel BMD | Myopia | -0.010 | 0.002 | -0.018 | 0.061 |
| rs7247412 | Heel BMD | Myopia | -0.012 | 0.002 | 0.021 | 0.072 |
| rs72810976 | Heel BMD | Myopia | -0.011 | 0.002 | 0.061 | 0.059 |
| rs72832241 | Heel BMD | Myopia | -0.019 | 0.002 | -0.016 | 0.070 |
| rs7290979 | Heel BMD | Myopia | 0.024 | 0.002 | 0.046 | 0.061 |
| rs7317781 | Heel BMD | Myopia | 0.015 | 0.002 | -0.022 | 0.065 |
| rs7368383 | Heel BMD | Myopia | 0.015 | 0.002 | 0.003 | 0.060 |
| rs737524 | Heel BMD | Myopia | 0.017 | 0.002 | -0.033 | 0.058 |
| rs7463837 | Heel BMD | Myopia | 0.012 | 0.002 | -0.022 | 0.067 |
| rs7484147 | Heel BMD | Myopia | 0.047 | 0.002 | 0.002 | 0.058 |
| rs7488974 | Heel BMD | Myopia | -0.051 | 0.002 | 0.082 | 0.061 |
| rs7527300 | Heel BMD | Myopia | 0.025 | 0.002 | 0.046 | 0.058 |
| rs7577569 | Heel BMD | Myopia | -0.013 | 0.002 | 0.019 | 0.060 |
| rs7582828 | Heel BMD | Myopia | 0.021 | 0.002 | 0.106 | 0.079 |
| rs7585120 | Heel BMD | Myopia | -0.018 | 0.002 | 0.041 | 0.073 |
| rs7599234 | Heel BMD | Myopia | 0.012 | 0.002 | -0.035 | 0.057 |
| rs760402 | Heel BMD | Myopia | -0.012 | 0.002 | 0.127 | 0.064 |
| rs7646519 | Heel BMD | Myopia | -0.015 | 0.002 | 0.005 | 0.063 |
| rs7661259 | Heel BMD | Myopia | 0.014 | 0.002 | 0.061 | 0.062 |
| rs7703751 | Heel BMD | Myopia | 0.024 | 0.002 | 0.010 | 0.076 |
| rs77112003 | Heel BMD | Myopia | -0.014 | 0.002 | 0.091 | 0.064 |
| rs772175 | Heel BMD | Myopia | -0.010 | 0.002 | 0.056 | 0.058 |
| rs7771496 | Heel BMD | Myopia | 0.014 | 0.002 | 0.121 | 0.061 |
| rs7778978 | Heel BMD | Myopia | -0.012 | 0.002 | -0.039 | 0.062 |
| rs7789880 | Heel BMD | Myopia | 0.027 | 0.002 | -0.036 | 0.064 |
| rs7800633 | Heel BMD | Myopia | 0.011 | 0.002 | -0.053 | 0.059 |
| rs7814941 | Heel BMD | Myopia | -0.021 | 0.002 | -0.019 | 0.076 |
| rs7818431 | Heel BMD | Myopia | -0.010 | 0.002 | 0.055 | 0.057 |
| rs785836 | Heel BMD | Myopia | -0.012 | 0.002 | 0.022 | 0.059 |
| rs7866211 | Heel BMD | Myopia | 0.025 | 0.002 | -0.011 | 0.062 |
| rs7898342 | Heel BMD | Myopia | -0.012 | 0.002 | 0.024 | 0.061 |
| rs7902719 | Heel BMD | Myopia | -0.011 | 0.002 | -0.053 | 0.059 |
| rs79730878 | Heel BMD | Myopia | 0.024 | 0.002 | 0.104 | 0.107 |
| rs8073697 | Heel BMD | Myopia | 0.014 | 0.002 | 0.104 | 0.065 |
| rs8114050 | Heel BMD | Myopia | 0.011 | 0.002 | 0.056 | 0.058 |
| rs8131741 | Heel BMD | Myopia | 0.019 | 0.002 | 0.071 | 0.059 |
| rs8180282 | Heel BMD | Myopia | -0.012 | 0.002 | -0.051 | 0.071 |
| rs8192803 | Heel BMD | Myopia | -0.011 | 0.002 | 0.089 | 0.060 |
| rs825453 | Heel BMD | Myopia | 0.016 | 0.002 | 0.049 | 0.058 |
| rs868127 | Heel BMD | Myopia | 0.017 | 0.002 | 0.090 | 0.067 |
| rs890074 | Heel BMD | Myopia | -0.012 | 0.002 | 0.106 | 0.059 |
| rs899631 | Heel BMD | Myopia | -0.018 | 0.002 | 0.031 | 0.060 |
| rs910405 | Heel BMD | Myopia | 0.013 | 0.002 | -0.075 | 0.062 |
| rs917441 | Heel BMD | Myopia | -0.029 | 0.002 | 0.062 | 0.061 |
| rs9257703 | Heel BMD | Myopia | 0.020 | 0.002 | -0.022 | 0.088 |
| rs9324890 | Heel BMD | Myopia | -0.012 | 0.002 | 0.054 | 0.063 |
| rs932828 | Heel BMD | Myopia | 0.032 | 0.002 | 0.031 | 0.060 |
| rs9447004 | Heel BMD | Myopia | 0.024 | 0.002 | 0.012 | 0.058 |
| rs947091 | Heel BMD | Myopia | -0.040 | 0.002 | -0.016 | 0.060 |
| rs9513510 | Heel BMD | Myopia | 0.035 | 0.002 | 0.017 | 0.061 |
| rs9521510 | Heel BMD | Myopia | 0.021 | 0.002 | 0.026 | 0.061 |
| rs9530279 | Heel BMD | Myopia | 0.019 | 0.002 | -0.037 | 0.066 |
| rs9545557 | Heel BMD | Myopia | -0.010 | 0.002 | 0.003 | 0.059 |
| rs957772 | Heel BMD | Myopia | 0.012 | 0.002 | -0.044 | 0.058 |
| rs960192 | Heel BMD | Myopia | -0.017 | 0.002 | -0.058 | 0.059 |
| rs9612225 | Heel BMD | Myopia | 0.011 | 0.002 | -0.095 | 0.072 |
| rs9725597 | Heel BMD | Myopia | -0.015 | 0.002 | -0.113 | 0.071 |
| rs9811191 | Heel BMD | Myopia | -0.014 | 0.002 | 0.005 | 0.058 |
| rs9896306 | Heel BMD | Myopia | 0.026 | 0.002 | 0.020 | 0.063 |
| rs9921222 | Heel BMD | Myopia | 0.026 | 0.002 | 0.004 | 0.058 |
| rs13423976 | FA-BMD | Myopia | -0.098 | 0.017 | -0.030 | 0.064 |
| rs6894139 | FA-BMD | Myopia | -0.089 | 0.016 | -0.078 | 0.058 |
| rs7776725 | FA-BMD | Myopia | 0.186 | 0.017 | 0.024 | 0.066 |
| rs10170839 | FN-BMD | Myopia | -0.059 | 0.008 | -0.013 | 0.058 |
| rs13194508 | FN-BMD | Myopia | -0.052 | 0.009 | -0.155 | 0.073 |
| rs1366594 | FN-BMD | Myopia | -0.079 | 0.008 | -0.078 | 0.058 |
| rs1485307 | FN-BMD | Myopia | 0.062 | 0.008 | -0.014 | 0.058 |
| rs1785493 | FN-BMD | Myopia | -0.045 | 0.008 | 0.057 | 0.071 |
| rs3779381 | FN-BMD | Myopia | 0.058 | 0.009 | 0.025 | 0.066 |
| rs436448 | FN-BMD | Myopia | -0.064 | 0.008 | -0.103 | 0.059 |
| rs7209460 | FN-BMD | Myopia | -0.051 | 0.008 | 0.033 | 0.062 |
| rs9478217 | FN-BMD | Myopia | -0.053 | 0.008 | -0.148 | 0.059 |
| rs11680288 | LS-BMD | Myopia | 0.054 | 0.009 | 0.027 | 0.058 |
| rs13046645 | LS-BMD | Myopia | -0.056 | 0.010 | -0.032 | 0.074 |
| rs1357651 | LS-BMD | Myopia | -0.068 | 0.009 | 0.045 | 0.065 |
| rs2220189 | LS-BMD | Myopia | 0.083 | 0.009 | -0.005 | 0.058 |
| rs2235811 | LS-BMD | Myopia | -0.054 | 0.009 | 0.027 | 0.058 |
| rs2291467 | LS-BMD | Myopia | -0.077 | 0.010 | 0.061 | 0.079 |
| rs401680 | LS-BMD | Myopia | -0.057 | 0.009 | -0.103 | 0.059 |
| rs6965122 | LS-BMD | Myopia | -0.062 | 0.009 | 0.098 | 0.063 |
| rs7807953 | LS-BMD | Myopia | 0.075 | 0.010 | 0.024 | 0.066 |
| rs894738 | LS-BMD | Myopia | -0.063 | 0.009 | -0.156 | 0.057 |
| rs9921222 | LS-BMD | Myopia | -0.053 | 0.009 | -0.004 | 0.058 |
| rs1037011 | TB-BMD | Myopia | -0.040 | 0.006 | 0.038 | 0.058 |
| rs10777212 | TB-BMD | Myopia | 0.045 | 0.006 | -0.065 | 0.068 |
| rs10788264 | TB-BMD | Myopia | -0.034 | 0.006 | 0.013 | 0.058 |
| rs10875906 | TB-BMD | Myopia | 0.051 | 0.007 | 0.020 | 0.061 |
| rs10901216 | TB-BMD | Myopia | -0.047 | 0.006 | 0.039 | 0.058 |
| rs11228240 | TB-BMD | Myopia | -0.083 | 0.007 | 0.123 | 0.076 |
| rs11745493 | TB-BMD | Myopia | 0.045 | 0.007 | -0.011 | 0.071 |
| rs11904127 | TB-BMD | Myopia | -0.032 | 0.006 | 0.029 | 0.058 |
| rs11934731 | TB-BMD | Myopia | -0.067 | 0.006 | 0.027 | 0.064 |
| rs11995824 | TB-BMD | Myopia | 0.068 | 0.006 | -0.019 | 0.058 |
| rs12534510 | TB-BMD | Myopia | -0.040 | 0.006 | -0.013 | 0.059 |
| rs13204965 | TB-BMD | Myopia | 0.062 | 0.007 | 0.163 | 0.074 |
| rs1452102 | TB-BMD | Myopia | -0.035 | 0.006 | 0.033 | 0.061 |
| rs1548607 | TB-BMD | Myopia | 0.036 | 0.007 | 0.065 | 0.061 |
| rs2252865 | TB-BMD | Myopia | -0.033 | 0.006 | 0.003 | 0.060 |
| rs2553773 | TB-BMD | Myopia | -0.037 | 0.006 | -0.035 | 0.058 |
| rs2873195 | TB-BMD | Myopia | -0.041 | 0.006 | 0.033 | 0.062 |
| rs34102936 | TB-BMD | Myopia | 0.047 | 0.006 | -0.073 | 0.062 |
| rs35199438 | TB-BMD | Myopia | -0.049 | 0.006 | 0.129 | 0.059 |
| rs3743347 | TB-BMD | Myopia | 0.052 | 0.007 | -0.051 | 0.076 |
| rs3801387 | TB-BMD | Myopia | -0.135 | 0.006 | -0.024 | 0.066 |
| rs447911 | TB-BMD | Myopia | 0.071 | 0.006 | 0.103 | 0.059 |
| rs55781332 | TB-BMD | Myopia | -0.055 | 0.007 | 0.043 | 0.063 |
| rs6029130 | TB-BMD | Myopia | 0.035 | 0.006 | -0.032 | 0.073 |
| rs6040063 | TB-BMD | Myopia | 0.036 | 0.006 | -0.027 | 0.058 |
| rs61837366 | TB-BMD | Myopia | 0.042 | 0.007 | -0.030 | 0.061 |
| rs633995 | TB-BMD | Myopia | 0.035 | 0.006 | -0.025 | 0.061 |
| rs6465511 | TB-BMD | Myopia | -0.074 | 0.006 | 0.097 | 0.063 |
| rs6557155 | TB-BMD | Myopia | -0.075 | 0.006 | -0.139 | 0.059 |
| rs6960249 | TB-BMD | Myopia | 0.033 | 0.006 | -0.069 | 0.058 |
| rs7105860 | TB-BMD | Myopia | -0.047 | 0.006 | -0.002 | 0.058 |
| rs725670 | TB-BMD | Myopia | -0.032 | 0.006 | -0.022 | 0.060 |
| rs73305797 | TB-BMD | Myopia | 0.042 | 0.007 | 0.006 | 0.075 |
| rs7548588 | TB-BMD | Myopia | -0.037 | 0.006 | 0.003 | 0.058 |
| rs757138 | TB-BMD | Myopia | -0.035 | 0.006 | 0.031 | 0.063 |
| rs7586085 | TB-BMD | Myopia | 0.053 | 0.006 | -0.024 | 0.057 |
| rs7728694 | TB-BMD | Myopia | -0.050 | 0.006 | -0.071 | 0.058 |
| rs7741085 | TB-BMD | Myopia | 0.042 | 0.006 | 0.006 | 0.059 |
| rs780096 | TB-BMD | Myopia | -0.031 | 0.006 | 0.074 | 0.059 |
| rs8047501 | TB-BMD | Myopia | 0.052 | 0.006 | 0.017 | 0.058 |
| rs8070128 | TB-BMD | Myopia | -0.039 | 0.006 | -0.088 | 0.058 |
| rs818427 | TB-BMD | Myopia | 0.034 | 0.006 | -0.016 | 0.061 |
| rs838721 | TB-BMD | Myopia | -0.031 | 0.006 | 0.042 | 0.058 |
| rs9972944 | TB-BMD | Myopia | 0.036 | 0.006 | 0.102 | 0.059 |
| rs9976876 | TB-BMD | Myopia | -0.038 | 0.006 | -0.078 | 0.058 |
| rs11102689 | RA | Myopia | 0.001 | 0.000 | -0.008 | 0.063 |
| rs17612712 | RA | Myopia | 0.001 | 0.000 | 0.054 | 0.068 |
| rs3104415 | RA | Myopia | 0.002 | 0.000 | 0.024 | 0.070 |
| rs4512588 | RA | Myopia | 0.001 | 0.000 | -0.255 | 0.535 |
| rs7331739 | RA | Myopia | 0.001 | 0.000 | 0.046 | 0.064 |

# IVs represents instruments variates; MR represents Mendelian randomization；FA-, FN-, LS-, TB-BMD represent forearm, femoral neck, lumbar spine and total body BMD respectively; RA represents rheumatic arthritis.
