# Supplementary material for: Rheumatoid arthritis and osteoporosis: a bi-directional Mendelian randomization study
Source: Aging (Albany NY). 2021 May 18;13(10):14109–30. doi: 10.18632/aging.203029 (PMC8202858; doi:10.18632/aging.203029)
Supplement: Supplementary Tables 3 and 4 [file aging-13-203029-s004.pdf]

SUPPLEMENTARY TABLES

Supplementary Table 3. MR results of negative control.

Exposure	Outcome	No. of IVs	MR results			
			Methods	Beta	Se	Pval
Heel BMD	Myopia	342	Inverse variance weighted	0.133	0.175	0.447
			MR Egger	0.197	0.357	0.583
			Weighted median	-0.082	0.292	0.779
			Weighted mode	-0.303	0.442	0.493
FA-BMD	Myopia	3	Inverse variance weighted	0.300	0.281	0.285
			MR Egger	-0.381	0.841	0.729
			Weighted median	0.208	0.319	0.514
			Weighted mode	0.162	0.329	0.671
FN-BMD	Myopia	14	Inverse variance weighted	0.814	0.410	0.047
			MR Egger	1.657	2.762	0.567
			Weighted median	0.616	0.474	0.194
			Weighted mode	0.521	0.624	0.428
LS-BMD	Myopia	14	Inverse variance weighted	0.088	0.408	0.829
			MR Egger	-0.909	3.288	0.790
			Weighted median	0.052	0.464	0.911
			Weighted mode	0.090	0.781	0.911
TB-BMD	Myopia	50	Inverse variance weighted	-0.090	0.197	0.647
			MR Egger	0.193	0.545	0.725
			Weighted median	-0.259	0.310	0.403
			Weighted mode	0.048	0.473	0.919
RA	Myopia	6	Inverse variance weighted	20.092	24.974	0.421
			MR Egger	8.850	52.780	0.878
			Weighted median	19.148	28.146	0.496
			Weighted mode	16.835	30.698	0.613

IVs represents instruments variates; MR represents Mendelian randomization; FA-, FN-, LS-, TB-BMD represent forearm, femoral neck, lumbar spine and total body BMD respectively; RA represents rheumatic arthritis.

Supplementary Table 4. IVs of RA for MR analyses in stage 2.

Exposure	Outcome	SNP	Effect allele	Other allele	GWAS Beta		GWAS P	
					Exposure	Outcome	Exposure	Outcome
RA	Heel BMD	rs11102689	A	T	6.50E-04	0.006	4.90E-06	0.006
		rs11687715	G	T	-5.48E-04	0.004	6.20E-06	0.015
		rs3104415	C	A	2.14E-03	-0.007	9.50E-65	0.000
		rs4318292	C	T	-5.68E-04	-0.001	2.20E-06	0.750
RA	FA-BMD	rs11102689	A	T	6.50E-04	0.011	4.90E-06	0.579
		rs11687715	G	T	-5.48E-04	0.021	6.20E-06	0.191
		rs4318292	C	T	-5.68E-04	-0.022	2.20E-06	0.165
		rs4512588	C	T	9.72E-04	-0.039	2.50E-08	0.301
		rs7331739	C	G	6.07E-04	0.013	4.10E-06	0.462
		rs741384	G	C	6.09E-04	0.023	4.70E-07	0.137
RA	FN-BMD	rs11102689	A	T	6.50E-04	0.024	4.90E-06	0.018
		rs11687715	G	T	-5.48E-04	0.011	6.20E-06	0.158
		rs3104415	C	A	2.14E-03	-0.017	9.50E-65	0.163
		rs4318292	C	T	-5.68E-04	-0.006	2.20E-06	0.448
		rs4512588	C	T	9.72E-04	-0.041	2.50E-08	0.082
		rs7331739	C	G	6.07E-04	-0.004	4.10E-06	0.601
		rs741384	G	C	6.09E-04	-0.002	4.70E-07	0.793
RA	LS-BMD	rs11102689	A	T	6.50E-04	0.011	4.90E-06	0.355
		rs11687715	G	T	-5.48E-04	0.015	6.20E-06	0.098
		rs3104415	C	A	2.14E-03	-0.006	9.50E-65	0.648
		rs4318292	C	T	-5.68E-04	0.009	2.20E-06	0.345
		rs4512588	C	T	9.72E-04	-0.006	2.50E-08	0.819
		rs7331739	C	G	6.07E-04	-0.001	4.10E-06	0.883
		rs741384	G	C	6.09E-04	0.014	4.70E-07	0.132
RA	TB-BMD	rs11102689	A	T	6.50E-04	0.066	4.90E-06	0.575
		rs11687715	G	T	-5.48E-04	0.002	6.20E-06	0.800
		rs3104415	C	A	2.14E-03	-0.005	9.50E-65	0.497
		rs4318292	C	T	-5.68E-04	-0.004	2.20E-06	0.478
		rs4512588	C	T	9.72E-04	0.011	2.50E-08	0.312
		rs7331739	C	G	6.07E-04	-0.011	4.10E-06	0.099
		rs741384	G	C	6.09E-04	0.005	4.70E-07	0.450

IVs represents instruments variates; MR represents Mendelian randomization; FA-, FN-, LS-, TB-BMD represent forearm, femoral neck, lumbar spine and total body BMD respectively; RA represents rheumatic arthritis.
